# Regional gene expression signatures are associated with sex-specific functional connectivity changes in depression

**DOI:** 10.1038/s41467-022-32617-1

**Published:** 2022-09-28

**Authors:** Aleksandr Talishinsky, Jonathan Downar, Petra E. Vértes, Jakob Seidlitz, Katharine Dunlop, Charles J. Lynch, Heather Whalley, Andrew McIntosh, Fidel Vila-Rodriguez, Zafiris J. Daskalakis, Daniel M. Blumberger, Conor Liston

**Affiliations:** 1 Department of Psychiatry, Weill Cornell Medicine, New York, NY USA; 2 Feil Family Brain and Mind Research Institute, Weill Cornell Medicine, New York, NY USA; 3 Krembil Research Institute and Centre for Mental Health, University Health Network, Toronto, ON USA; 4 Department of Psychiatry, University of Toronto, Toronto, ON USA; 5 Department of Psychiatry, University of Cambridge, Cambridge, UK; 6 Department of Child and Adolescent Psychiatry and Behavioral Science, Children’s Hospital of Philadelphia, Philadelphia, PA USA; 7 Department of Psychiatry, University of Pennsylvania, Philadelphia, PA USA; 8 Center for Clinical Brain Sciences, University of Edinburgh, Edinburgh, UK; 9 Non-Invasive Neurostimulation Therapies Lab and Department of Psychiatry, University of British Columbia, Vancouver, BC USA; 10 Department of Psychiatry, University of California, San Diego, California USA; 11 Temerty Centre for Therapeutic Brain Intervention, Centre for Addiction and Mental Health, Toronto, ON USA

**Keywords:** Depression, Depression

## Abstract

The neural substrates of depression may differ in men and women, but the underlying mechanisms are incompletely understood. Here, we show that depression is associated with sex-specific patterns of abnormal functional connectivity in the default mode network and in five regions of interest with sexually dimorphic transcriptional effects. Regional differences in gene expression in two independent datasets explained the neuroanatomical distribution of abnormal connectivity. These gene sets varied by sex and were strongly enriched for genes implicated in depression, synapse function, immune signaling, and neurodevelopment. In an independent sample, we confirmed the prediction that individual differences in default mode network connectivity are explained by inferred brain expression levels for six depression-related genes, including *PCDH8*, a brain-specific protocadherin integral membrane protein implicated in activity-related synaptic reorganization. Together, our results delineate both shared and sex-specific changes in the organization of depression-related functional networks, with implications for biomarker development and fMRI-guided therapeutic neuromodulation.

## Abstract (web summary)

The neural substrates of depression may differ by sex. Here the authors show that depression is associated with distinct brain connectivity changes in men and in women that are explained by sex-specific transcriptomic signatures involving genes previously implicated in synapse function, immune signalling, and depression risk.

## Introduction

Depression (major depressive disorder) is a highly heterogeneous psychiatric syndrome with a weak correspondence to its biological substrates. In large-scale epidemiological studies, the lifetime incidence of depression is two-fold higher in women, such that female sex is among the strongest known risk factors^1^, but the mechanisms underlying sex differences in depression are poorly understood. Modest but statistically significant differences are evident in depression-related effects on brain structure^2,3^ and in the types of symptoms that tend to occur in men versus women^1,4^. For example, comorbid substance use disorders are more common in men with depression, while anxiety symptoms are more common in women with depression^4–7^. Recent studies have also identified sexually-dimorphic gene expression signatures^8,9^, suggesting that sex-specific mechanisms may contribute to depression at both a molecular and a systems level.

Functional magnetic resonance imaging (fMRI) has become one of the most important tools for studying depression neurobiology in humans. Multiple studies have identified alterations in resting state functional connectivity (rsFC) in the default mode network (DMN), limbic areas, and other depression-related networks^10–14^, which have potential utility for predicting treatment response and as biomarkers of remission^15–18^. Default mode network (DMN) hyperconnectivity is among the most consistently replicated findings in rsfMRI studies of depression^10,13,15,19,20^, and may also be involved in other neuropsychiatric disorders^21^, including dementia^22^, schizophrenia^23^, and autism^24^, among others. It has been implicated in anxious rumination and negative biases in self-referential processing^21,25^ and is a potential therapeutic target for neurostimulation and biofeedback interventions^26,27^. Other studies have identified abnormal interactions between the DMN and cognitive control networks, which may contribute to cognitive control deficits^13,26,28^. However, while many studies have observed DMN hyperconnectivity in depression, others have not^29,30^, and at least one recent large-scale analysis found a reduction in rsFC in this network^11^. Surprisingly, relatively few studies to date have tested for sex-specific effects on rsFC in depression, due in part to power constraints. Whether depression is associated with distinct patterns of circuit dysfunction in women versus men remains unclear.

The mechanisms that give rise to rsFC effects in depression are also not well understood. Large-scale genome-wide association studies involving >100,000 subjects have identified numerous genetic risk variants, but they also underscore a highly polygenic inheritance pattern, in which each variant confers only a small increase in risk^31–35^. How dozens or even hundreds of genetic variants interact to regulate depression pathophysiology is unknown. Undoubtedly, the mechanisms are complex and multifactorial, but converging evidence from other areas of study support the hypothesis that spatial variation in the brain transcriptome could influence functional connectivity^36,37^. For example, recent reports indicate that regional differences in gene expression may contribute to the neuroanatomical distribution of morphometric and connectivity effects in neuropsychiatric disease states including autism and schizophrenia^38–41^. In at least one recent report, the expression of astrocyte- and interneuron-related genetic markers was correlated with spatial patterns of abnormal functional connectivity associated with depression and negative affect^42^. Whether regional differences in gene expression explain the specific connections and networks exhibiting rsFC changes in depression, and whether these genetic correlates differ by sex, is unknown.

Here, we aimed to test the hypothesis that 1) depression is associated with sex-dependent patterns of abnormal connectivity; 2) some depression-related genes may influence pathophysiology by modulating functional connectivity in depression-related networks; and 3) different gene expression modules may be important in men and women with depression. Leveraging a large-scale rsfMRI dataset comprising scans from over 500 individuals with depression and healthy controls, we first tested whether depression is associated with rsFC effects that vary by sex, focusing on the default mode network—which has been consistently implicated in depression and anxious rumination in multiple fMRI studies^19,43–45^—and on five regions of interest that exhibit sexually dimorphic transcriptional effects in depression^8^. Our analyses identified rsFC effects shared by both men and women, as well as multiple sex-specific abnormalities in the default mode network and in all five ROIs, to varying degrees. They include multiple targets for therapeutic neuromodulation interventions that are informed by functional mapping and may benefit from accounting for sex differences. Finally, using microarray data from two datasets—the Allen Human Brain Atlas^46^ and Brainspan^47^—we showed that regional differences in the expression of depression-related genes explain the neuroanatomical distribution of circuits and networks exhibiting altered rsFC in men and women with depression. Bioinformatic analyses showed that these gene sets are enriched for depression-related genes, GWAS-confirmed risk variants, and genes implicated in synapse function and immune signaling. They also identified specific genes that are transcriptionally altered in depression, regulated by GWAS-confirmed risk variants, and predict individual differences in functional connectivity in men and women with depression.

## Results

### Shared and sex-specific rsFC effects in a depression-related network

Default mode network (DMN) hyperconnectivity is a consistently replicated finding in many^10,13,15,19,20^, but not all^29,30^, rsfMRI studies of depression, and a potential therapeutic target for neurostimulation^26,27^. Importantly, none of these previous reports were designed to investigate sex differences. Thus, we began by implementing an unbiased, whole-brain quantification of rsFC abnormalities within the DMN and between the DMN and other functional networks in depression. This analysis was implemented in a rsfMRI dataset comprising 371 patients (*n* = 223 women) with treatment-resistant depression (actively depressed, moderate or severe intensity, mean HAMD in males = 23.2 ± 4.8, mean HAMD in females = 23.8 ± 4.3) and 182 healthy control subjects (*n* = 103 women) acquired on three scanners (see Supplementary Table 1 and Supplementary Methods for additional details, including preprocessing, quality control, and correction for scanner effects). BOLD signal time series were extracted for 360 cortical areas defined by an extensively validated functional parcellation^48^ and 19 subcortical regions^49^. These functional parcels were used to generate rsFC matrices between 77 default mode network ROIs and the rest of the brain.

**Fig. 1:**
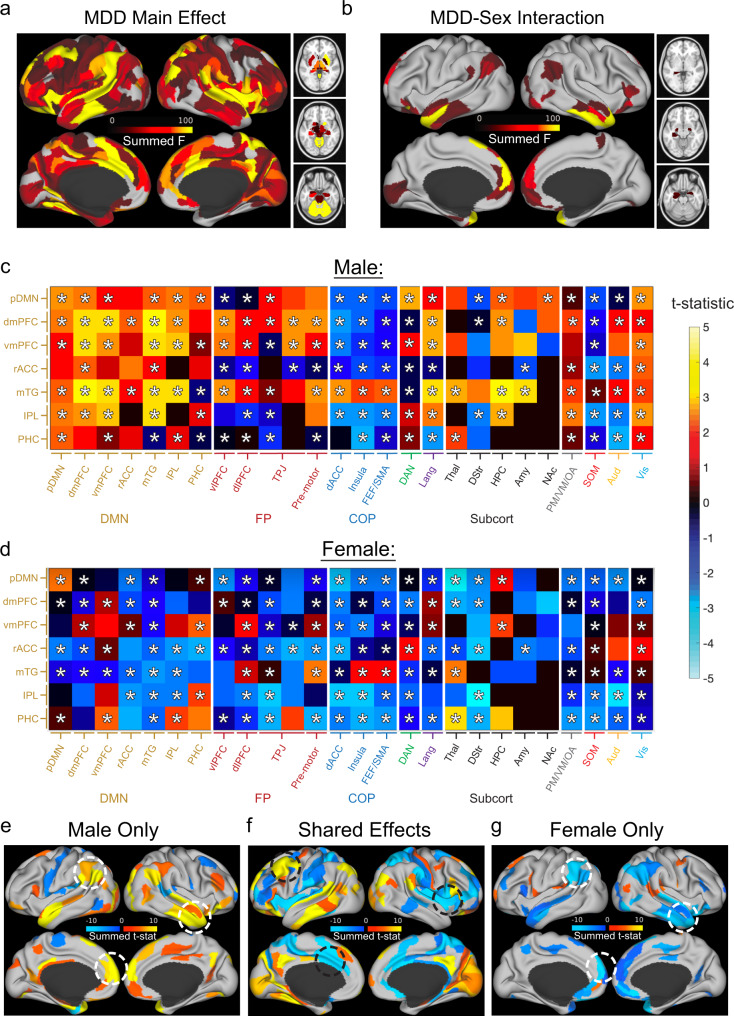
Shared and sex-specific rsFC abnormalities in a depression-related network. **a**, **b** Color maps representing the neuroanatomical distribution of significant (FDR *q* < 0.05) F-statistics from 2-way ANOVA for rsFC features connecting 77 default mode network (DMN) nodes and the rest of the brain in *n* = 371 patients with major depressive disorder (MDD) (*n* = 223 women) vs. *n* = 182 healthy controls (*n* = 103 women). The neuroanatomical distribution of significant main effects of MDD (**a**) and MDD-by-sex interactions (**b**) are summarized for each functional parcel by summing across the significant effects in the 77 DMN nodes and plotting the result on a brain surface and accompanying subcortical image. **c**, **d** Summary t-statistics from posthoc t-tests comparing male (**c**) and female (**d**) MDD patients vs. healthy controls, summarized by grouping neuroanatomically adjacent Glasser HCPMM1 parcels within each network. Colors in each tile represent the average t-statistic for all rsFC features in a given region that exceed a threshold of *p* < 0.05 uncorrected. Positive t-statistics denote increased rsFC in MDD. Regions with at least one significant (FDR *q* < 0.05) MDD effect or MDD-Sex interaction effect in the ANOVA depicted in (**a**, **b**) are demarcated with an asterisk. **e**–**g** Color maps representing the neuroanatomical distribution of the significant post-hoc t-test results depicted in (**c**, **d**), summed as in (**a**, **b**). rsFC features that were significantly abnormal in (**e**) men with depression, **g** women with depression, or (**f**) both men and women are plotted separately. White dashed circles denote sex-specific DMN effects discussed in the main text. Black dashed circles denote shared effects discussed in the main text. DMN default mode network, pDMN posterior DMN (precuneus, posterior cingulate), dmPFC dorsomedial prefrontal cortex, vmPFC ventromedial PFC, rACC rostral anterior cingulate, mTG medial temporal gyrus, vlPFC ventrolateral PFC, IPL inferior parietal lobule, PHC parahippocampal cortex, FP fronto-parietal network, dlPFC dorsolateral PFC, TPJ temporo-parietal junction, COP cingulo-opercular network, dACC dorsal anterior cingulate, FEF/SMA frontal eye field/supplementary motor area, DAN dorsal attention network, Lang language network, Subcort subcortical ROIs, PM/VM/OA posterior multimodal, ventral multimodal, orbito-affective networks, SOM somatosensory-moto network, Aud auditory network, Vis visual network.

Our analysis revealed widespread main effects of depression on DMN connectivity spanning most of the brain, with the most significant effects localized to the anterior cingulate, dorsomedial prefrontal cortex, lateral prefrontal cortex, and insula (Fig. 1a), in agreement with previous work^10,13,20,43^. We also detected significant main effects of sex (Supplementary Fig. S1) and sex-by-depression interactions (Fig. 1b) that have not been extensively examined in previous work and were localized primarily to a large area of the medial prefrontal cortex and the temporal pole. Post-hoc contrasts within each sex identified multiple effects shared by both sexes (Fig. 1c, d, f), including reduced connectivity with anterior cingulate and insular nodes of the cingulo-opercular control network and increased connectivity with the specific visual network, temporal language network, and dorsolateral prefrontal areas. However, many effects were sex-specific (Fig. 1e, g), and the strongest effects were driven by men. Of note, hyperconnectivity within the DMN—among the most extensively studied correlates of depression^10,13,20,43^ —occurred almost exclusively in men in our sample and was driven by the temporal pole, middle temporal gyrus, orbitofrontal cortex, and dorsomedial prefrontal cortex (Fig. 1e). In contrast, in women with depression, rsFC was modestly decreased in most of these areas (Fig. 1g). We also found that these effects were not driven by scanner-related artifacts: comparable effects were observed in an analysis restricted to data from *n* = 371 patients with depression (*n* = 223 women) and *n* = 85 healthy controls (*n* = 52 women) acquired on a single scanner (Supplementary Fig. S2), validating our approach to controlling for scanner-related confounds (see Supplementary Methods). Of note, men and women with depression in our sample did not differ with respect to symptom severity (Supplementary Table 1), which indicates that the differing effects in men and women were not driven simply by severity. Instead, different connectivity abnormalities may contribute to depression in males vs. females, with DMN hyperconnectivity being more important in males and other features being more important in females (see below).

**Fig. 2:**
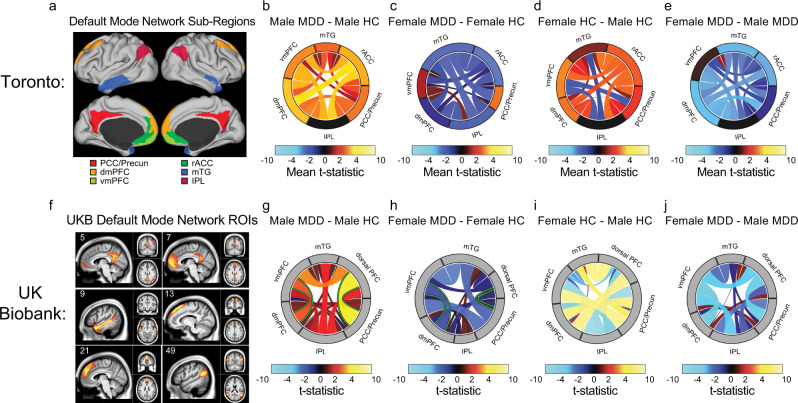
Sex-dependent effects of depression on DMN connectivity in two independent samples. **a** Default mode network (DMN) ROIs in the Glasser parcellation used in our primary analyses. **b**–**e** Circle plots (facilitating comparisons with the UK Biobank parcellation in [**f–j**]) depicting rsFC effects for six DMN subregions comparing four groups of subjects: healthy men (*n* = 79), men with MDD (*n* = 148), healthy women (*n* = 103), and women with MDD (*n* = 223). Colors and band thickness scale with the mean of all significant (unadjusted *p* < 0.05) t-statistics. Warm colors denote increased connectivity in MDD patients compared to controls (**b**, **c**) or in women compared to men (**d**, **e**). dmPFC dorsomedial prefrontal cortex, vmPFC ventromedial prefrontal cortex, rACC rostral anterior cingulate, PCC/Precuneus posterior cingulate and precuneus, mTG medial temporal gyrus, IPL inferior parietal lobule. **f** Six UK BioBank ICA components comprising the DMN are depicted in sagittal, coronal, and axial brain images. The component numbers in the upper left of each image specify the ICA component as defined in the UK Biobank ICA parcellation, available at https://www.fmrib.ox.ac.uk/ukbiobank/. Component 5 = posterior cingulate/precuneus (PCC/Precun); 7 = ventromedial prefrontal cortex (vmPFC); 9 = medial temporal gyrus (mTG); 13 = dorsomedial PFC (dmPFC); 21 = dorsal PFC; and 49 = inferior parietal lobule (IPL). **g–j** Circle plots (facilitating comparisons with the Glasser parcellation in [**a–e**]) depicting rsFC effects for six DMN sub-regions comparing four groups of subjects as in (**b–e**): healthy men (*n* = 1907), men with probable MDD who also reported severe MDD symptoms at the time of their scan (*n* = 28), healthy women (*n* = 1773), and women with probable MDD who also reported severe MDD symptoms at the time of their scan (*n* = 81). Warm colors denote increased connectivity in symptomatic UKB subjects with probable MDD compared to controls (**g**, **h**) or in women compared to men (**I**, **j**). Effects showing a significant MDD-by-sex interaction effect (FDRq < 0.05) are highlighted in green. Note that we used circle plots to represent the results in order to facilitate comparisons between the two parcellations. In the Glasser parcellation, each region included multiple functional parcels, so each band represents the mean of all significantly different connections between a given pair of regions. In the UKB parcellation, each region is represented by just one ICA-based functional parcel, so each band represents a single t-statistic between a given pair of regions.

To further understand the relationship between depression status, sex, and DMN connectivity, we plotted post-hoc t test results for effects of depression within each sex and for effects of sex within each diagnosis (Fig. 2a–e). We found that rsFC within the DMN was elevated in both men with depression (Fig. 2b) and healthy control women (Fig. 2d), compared to healthy men with no history of depression. That is, the relative paucity of effects of depression on connectivity within the DMN in women was due to the fact that DMN connectivity was increased in healthy control women relative to healthy control men. These effects were evident throughout multiple sub-regions of the DMN, including anterior (dorsomedial prefrontal, rostral anterior cingulate), posterior (precuneus, posterior cingulate), and lateral areas (middle temporal, inferior parietal).

DMN hyperconnectivity is one of the most extensively studied findings in fMRI studies of depression^10,13,20,43^, but most studies to date have treated sex as a covariate and were not designed to investigate interactions between sex and depression. Furthermore, the results above were obtained from subjects with treatment-resistant depression of moderate-to-severe intensity, who may differ from patients with other types of depression. To evaluate the extent to which these results generalize to other subject samples scanned and preprocessed under different conditions, we repeated this analysis in *n* = 3789 subjects (*n* = 109 of whom had symptomatic MDD) from the UK Biobank database using DMN nodes derived from an ICA-based functional parcellation implemented by UK Biobank investigators (see Fig. 2f and Supplementary Methods). Again, male subjects who self-reported significant depressive symptoms at the time of their scan (*n* = 28) showed significant DMN hyperconnectivity compared to healthy control men (*n* = 1907), but female subjects did not (*n* = 81 with self-reported depressive symptoms, *n* = 1773 healthy controls) (Fig. 2g–j). As in our primary sample, rsFC in most DMN sub-regions was also elevated in healthy control women compared to healthy men without depression (Fig. 2i). A similar pattern was observed in a larger cohort of UK Biobank subjects who were classified by UK Biobank investigators as having a probable MDD history based on a previously established UK Biobank protocol^50^ (Supplementary Fig. S3, *n* = 1458 subjects [921 female]) but were not necessarily symptomatic at the time of their rsfMRI scan. Together, these results show that within-network DMN hyperconnectivity is correlated with depression status, but in multiple subject cohorts recruited and scanned under differing conditions, this effect is driven by men. Within-network DMN hyperconnectivity also occurs in a large number of women without any history of depression—a finding with implications for future studies of sex differences in depression vulnerability and interpreting biomarkers of remission^15^.

### Sex-dependent rsFC effects in five transcriptomic regions of interest

Large-scale genome-wide association studies have identified at least 223 genetic variants associated with 426 genes conferring risk for depression^31–33,51,52^. It is unclear how they interact to modulate depression pathophysiology, but converging evidence from other disorders support the hypothesis that common genetic variants and regional differences in gene expression can influence functional network organization^36,38–42^. Recent RNA sequencing studies have identified sexually dimorphic gene expression changes related to depression in five key brain regions: subgenual cingulate cortex (Brodmann Area [BA] BA25), dorsal prefrontal cortex (BA8/9), nucleus accumbens (NAc), orbitofrontal cortex (BA11), and anterior insula^8^. All five ROIs have been implicated in multiple fMRI studies of depression^43,53–58^, but sex effects have not been examined. Whether regional differences in gene expression explain the neuroanatomical distribution of connectivity changes in depression is also unknown. Motivated by these findings, we first tested whether these five regions with sex-specific transcriptomic effects also exhibit connectivity effects that vary by sex.

**Fig. 3:**
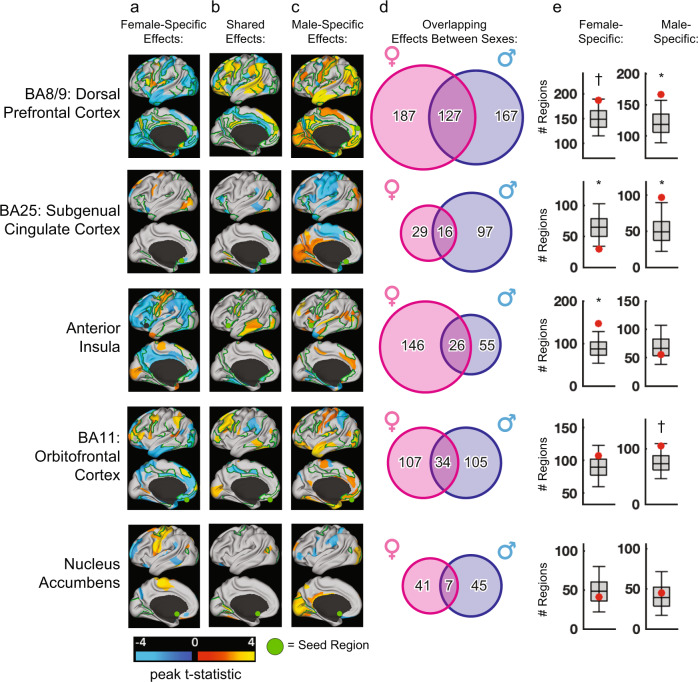
Sex-specific rsFC changes in five transcriptomic regions of interest. **a**–**c** Color maps of depressed vs healthy t-statistics showing the neuro-anatomical distribution of rsFC abnormalities that are (**a**) female-specific, **b** shared between sexes, or (**c**) male-specific in five transcriptomic seed ROIs labeled in rows, depicting the peak t-statistic across both hemispheres for each functional parcel. Parcels with significant (FDR *q* < 0.05) main effects of MDD or MDD-by-sex interactions are outlined in green and presented in detail in Supplementary Fig. S4. To illustrate how sex-specificity is not driven by stringent thresholding, we also plot nominally significant effects (unadjusted *p* < 0.05) in the parcels not outlined in green. **d** Venn diagrams depicting the number of sex-specific vs. shared connectivity effects at a liberal threshold (unadjusted *p* < 0.05) at each seed ROI. Sex specificity was even more pronounced at more stringent thresholds (Supplementary Fig. S4c, d). **e** Boxplots depicting the distribution of female- (left) and male-specific (right) connectivity effects in a null model with sex labels randomly permuted 1000 times. Red dots represent the empirically observed number of sex-specific effects from (**d**). † = *p* < 0.1; * = *p* < 0.05; ** = *p* < 0.01.

We found distinct effects of depression on rsFC in men versus women in all five ROIs, to varying degrees (Fig. 3a–c). Sex-specific effects were more common than shared effects in all five ROIs (Fig. 3d). Only dorsal prefrontal cortex (BA8/9) showed a large number of shared effects (Fig. 3d), and most effects were strikingly different in men vs. women, often in opposing directions (Fig. 3a–c), with hyperconnectivity predominating in men and hypoconnectivity predominating in women. In the subgenual cingulate cortex—a therapeutic target for deep brain stimulation and transcranial magnetic stimulation^17,18,59^—depression effects on rsFC were also highly divergent in men and women. In men with depression, subgenual cingulate connectivity was increased with posterior cingulate areas of the DMN and decreased with the anterior insula, temporal pole, and lateral prefrontal cortex, among other areas (Fig. 3c). None of these effects were observed in women (Fig. 3a). In the anterior insula, orbitofrontal cortex, and nucleus accumbens, the vast majority of observed effects were specific to one sex, and only a small proportion (7.5–13.8%) were shared by both sexes (Fig. 3d). Of note, there is increasing interest in using subgenual cingulate connectivity with the dorsolateral prefrontal cortex to inform prefrontal target selection for therapeutic TMS^17,18^. The nucleus accumbens, orbitofrontal cortex, and dorsomedial prefrontal cortex are also potential direct or indirect targets for TMS, DBS, or both^60–63^. One implication of these results is that fMRI-guided target selection strategies might benefit from accounting for sex.

Because the likelihood of observing effects specific to one sex could depend on the number of significantly altered connections and on effect size, we tested whether the number of sex-specific effects was larger than expected by chance compared to shuffled data with randomly permuted sex labels. We found strong evidence for sex-specific effects in three of the five regions (Fig. 3e). Mass univariate ANOVA analyses showed a similar pattern (Supplementary Fig. S4a–d): main effects of effects and significant sex-by-diagnosis interactions were most pronounced in dorsal prefrontal cortex (BA8/9). We also observed nominally significant sex-by-diagnosis interactions in the other four regions, but they did not survive FDR correction for multiple comparisons at the whole-brain level. Finally, we performed an unbiased whole-brain search to evaluate the degree to which sex-specificity in depression effects in these five ROIs was also observed in other brain areas. This analysis identified additional candidate regions with strong evidence of effects that differed by sex (Supplementary Fig. S4e). Together, these findings indicate that men and women with depression exhibit distinct patterns of abnormal functional connectivity in five transcriptomic ROIs that are therapeutic targets and that show divergent gene expression changes in depression^8^.

### Transcriptional signatures underlying sex-specific rsFC effects

**Fig. 4:**
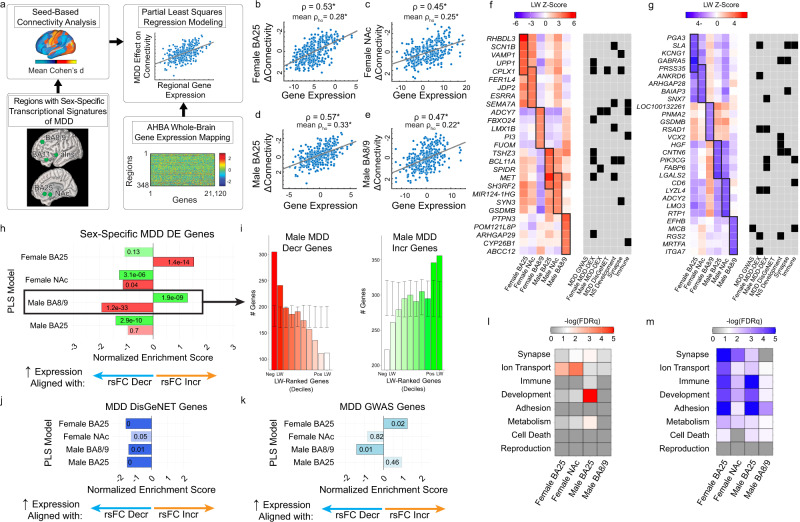
Distinct transcriptional signatures underlying sex-dependent rsFC effects in depression. **a** Schematic representing partial least squares regression (PLS-R) analysis for uncovering spatial relationships between regional gene expression patterns and rsFC abnormalities in five transcriptomic ROIs (BA25, NAc, BA11, BA8/9, aIns). **b–e** PLS-R identified multivariate spatial correlations between gene expression and rsFC effects that were statistically significant and reproducible in cross-validation in four models: female BA25 (**b**), female NAc (**c**), male BA25 (**d**), and male BA8/9 (**e**). In each scatterplot, individual data points represent gene expression and rsFC effects for a given brain region, projected into a space defined by the first PLS component. The correlation for the first PLS component (ρ) and the mean correlation in held-out data across 10 iterations of 10-fold cross validation (ρ_ho_) are depicted at the top. Statistical significance was evaluated compared to shuffled predictor data (spin test + FDR correction; see Online Methods). * = FDR *q* < 0.05. **f**, **g** Heatmaps depicting the loading weight (LW) Z-scores for the top 5 genes (in rows) with the strongest positive LWs (**f**) and the strongest negative LWs (**g**) in the PLS models depicted in (**b–e**), shown in columns. For comparison purposes, we also plot the corresponding models for the same three ROIs in the opposite sex, which were nominally significant (*P*_rand_ < 0.05, see Supplementary Fig. 5). Black tiles in the gray tables to the right of each heatmap denote membership of corresponding genes (labeled in rows) in selected gene sets relevant to MDD, as defined by a recent large-scale depression GWAS^52^ (“MDD GWAS”), the DisGeNet platform, or an RNA-seq study identifying differentially expressed genes in brain tissue in MDD patients (“MDD DEX”)^8^, or implicated in neurodevelopment, synapse function, or immune signaling as per the corresponding Gene Ontology Biological Process terms. **h** fGSEA results for differentially expressed genes in depression. In all four PLS models, genes predicting the spatial distribution of connectivity abnormalities in MDD are enriched for genes that show increased (green) or decreased (red) expression in a previously published RNA-seq analysis of brain tissue donated by MDD subjects of the corresponding sex^8^. fGSEA-generated normalized enrichment scores (x-axis), p-values (plotted in each bar), and adjusted p-values (darkened bar color if FDR *q* < 0.05) are plotted. Negative enrichment scores denote enrichment among genes with negative LWs in the PLS regression model, and positive enrichment scores denote enrichment among genes with positive LWs. **i** Histograms depicting the number of genes (y-axis and qualitative color gradient) with increased (left, green) or decreased (right, red) expression in males with MDD in each loading weight decile of our PLS-R ranked gene list for the male BA8/9 model. Error bars signify the 95% confidence interval of expected number of genes per decile in 10,000 simulations using random gene sets. **j**, **k** fGSEA results for depression-related risk genes as defined in DisGeNet database (**j**) and genes whose expression in nervous system tissue is modulated by significant SNPs from the most recent large-scale depression GWAS^52^ (**k**). **l**, **m** Gene Ontology (GO) analysis identified enrichments for GO Biological Process terms containing the corresponding phrases (listed in rows) at the (**l**) top and (**m**) bottom of LW-ranked gene lists in the four PLS models listed in (**b–e**). Significance of GO enrichment (i.e. -log(FDRq)) depicted in color scale.

The results above show that men and women with depression exhibit differing connectivity effects in five key brain regions that also featured sexually dimorphic transcriptomic effects in a previous report^8^. In all five regions, specific subsets of connections were altered in depression, and the spatial pattern of affected connections differed in men and women. We hypothesized that regional differences in the expression of depression-related genes—i.e. genes with altered expression in post-mortem brain tissue and genes linked to depression risk and pathophysiology—would explain the neuroanatomical distribution of connectivity effects in men and women with depression. To test this, we mapped normative regional gene expression profiles for 21,120 microarray probes in the Allen Human Brain Atlas^46^, which provides regional gene expression data from *n* = 6 subjects (*n* = 1 woman, ages 24–57), to the Glasser HCPMM1 functional parcellation used in Figs. 1–3^48^ using established methods. (Of note, our analysis was designed to detect genes with strong *regional* differences in expression that might contribute to the spatial distribution of connectivity effects in the depressed brain, regardless of whether those genes exhibit sex-biased expression, which could not be characterized in AHBA. As described below, we also replicated key findings using a second gene expression atlas, Brainspan^64,65^, acquired from *n* = 8 individuals with balanced sex composition [4 females], ages 18–40, but profiling a smaller number of brain regions.) Among these 21,120 genes were 4571 genes showing abnormal expression that was specific to men with depression as defined in a previous transcriptomic study^8^ and 4766 genes with abnormal expression that was specific to women with depression. We used partial least squares regression (PLS-R)^66^ to identify combinations of genes whose regional expression patterns best explained the neuroanatomical distribution of depression-related connectivity effects for each of the five seed ROIs depicted in Fig. 3, examining men and women separately (Fig. 4a). To test whether PLS-R identified reproducible gene-connectivity associations, we performed 10 iterations of 10-fold cross validation to estimate model performance in held-out data.

We found that regional differences in depression-related gene expression were spatially correlated with the neuroanatomical distribution of connectivity effects most reliably in both training data and held-out data in three ROIs (Fig. 4b–e; Supplementary Fig. S5, Supplementary Table 2): the subgenual cingulate cortex (BA25) in both females and males, the nucleus accumbens (NAc) in females only, and the dorsal prefrontal cortex (BA8/9) in males only. We evaluated the statistical significance of these associations using a spatially aware null permutation test (or “spin test”)^67^ that preserves local homogeneity in brain transcriptional data (further details provided in Supplementary Methods). This approach revealed significant associations between gene expression and connectivity abnormalities for six of ten models tested (Supplementary Fig. S5): dorsal prefrontal cortex (BA8/9) in men, anterior insula in women, NAc in women, BA11 in women, and BA25 in women and men. In four of these models involving three ROIs (male and female BA25, female NAc, and male BA8/9), gene-connectivity associations were reproducible in held-out data (100 iterations of PLS-R using random 90% subsamples of subjects), with statistically significant correlations in held-out subjects (Fig. 4b–e). Although we focus here on the most robust results in these four models, we also observed nominally significant correlations for all ten models (Supplementary Fig. S5), in a commonly used, randomly shuffled permutation testing framework^68–70^. Together, these results show that regional differences in gene expression explain the neuroanatomical distribution of abnormal rsFC in men and women with depression, most reliably in the subgenual cingulate (BA25), dorsal prefrontal cortex (BA8/9), and nucleus accumbens.

To understand the degree to which distinct gene sets explained connectivity abnormalities in these ROIs in men and women with depression, we identified the most important genes in each model by ranking the 21,120 genes by their PLS loading weight (their contribution to predicting connectivity abnormalities seeded from each ROI). Genes with the highest positive loading weights, which showed greater expression in cortical areas exhibiting abnormally increased connectivity in depression, are depicted in Fig. 4f. Two patterns were evident. First, within each sex, genes with the highest positive loading weights in the subgenual cingulate cortex (BA25) model also had high positive loading weights in the nucleus accumbens model, but not in the dorsal prefrontal cortex (BA8/9) model, where connectivity abnormalities were explained by different genes. Second, the most important genes in each model tended to vary by sex: that is, genes with the highest positive loading weights for a given ROI in women tended to have lower loading weights for the same ROI in men, and vice versa, especially for BA25 and BA8/9. The same two patterns were evident for genes with the strongest negative loading weights (Fig. 4g), which were highly expressed in cortical areas exhibiting abnormally decreased connectivity in depression. We confirmed these patterns quantitatively and showed that they were not restricted to the top-ranked genes in each model by testing for correlations between the full 21,120-gene loading weight vectors for each model (Supplementary Fig. S6). Qualitatively, we also found that a majority of the most important genes in these models have been previously implicated in depression, nervous system development, synapse function, or immune signaling (Fig. 4f, g).

Next, we performed three analyses to more directly test the hypothesis that genes related to depression or sex are important predictors of the neuroanatomical distribution of abnormal connectivity in men and women with depression. First, we used fast gene set enrichment analyses (fGSEA)^71,72^ to test whether genes exhibiting sex-specific transcriptional effects in a recent postmortem RNA sequencing study^8^ were overrepresented among highly weighted genes in the PLS-R models of the corresponding sex in our dataset. We found that in all four PLS-R models, transcriptomic signatures of depression from the corresponding sex were significantly over-represented among genes with the largest loading weights (Fig. 4h), with the most significant enrichments occurring in the male BA8/9 PLS-R model (Fig. 4i). Second, we used the same approach to test for enrichment of genes associated with depression-related risk variants. We found that genes modulated by GWAS-confirmed risk variants^31–33,51,52^ and genes implicated in MDD via the DisGeNET^73^ platform both showed significant enrichments in our PLS-R models (Fig. 4j, k, Supplementary Fig. S7). Finally, we found that genes previously shown to have sex-biased brain expression also showed significant enrichment among the most negative-loading genes in all four PLS-R models (Supplementary Fig. S8)—effects that were especially pronounced for genes exhibiting sex-biased expression early in life.

To delineate the specificity of these enrichments, we tested for over-representation of risk genes for other diseases, including other neuropsychiatric disorders (Supplementary Fig. S9). There was no evidence of enrichment for genetic risk variants associated with prostate cancer, colon cancer, breast cancer, or autism in any model, or for hypertension or type-2 diabetes in the female models. However, genes explaining connectivity abnormalities in BA8/9 in men were enriched for genetic variants conferring risk for hypertension and type-2 diabetes—an unexpected but interesting result in light of work linking depression and the metabolic syndrome^74,75^. Among other neuropsychiatric disorders, genetic risk variants for bipolar disorder, schizophrenia, and Alzheimer’s disease were significantly over-represented in one or more models. Next, we used standard bioinformatic approaches to evaluate whether the most important genes in each model implicate specific biological functions. Gene ontology (GO) analysis showed that genes with the strongest positive or negative loading weights were enriched for pathways associated with synaptic transmission, ion channels, immune signaling, and development (Fig. 4l, m), and that the most highly prioritized biological functions varied by region and sex (Supplementary Fig. S10).

### Validating and replicating transcriptomic correlates of abnormal connectivity in depression

Together, the results above indicate that connectivity abnormalities and their most important transcriptomic correlates differ in men and women with depression, and they support the hypothesis that at least some genetic risk variants contribute to depression pathophysiology by modulating functional connectivity. However, an important limitation of these findings is that they are derived from post-hoc correlational analyses involving just one neuroimaging dataset and one normative gene expression atlas. Thus, we implemented three analyses aimed at replicating and validating our findings and testing key predictions, leveraging additional datasets.

First, we sought to replicate our gene set enrichment findings using the Brainspan gene expression atlas, mapping 20,287 gene expression probes for 30 brain regions (15 per hemisphere, acquired from *n* = 8 individuals, 4 females, 4 males, ages 18–40) to the Glasser HCPMM1 functional parcellation, as described above for AHBA. Although the Brainspan atlas profiles a smaller number of brain regions than the AHBA, it has the advantage of a more balanced sex composition, enabling us to examine male and female gene expression patterns separately. As above, we used partial least squares regression to identify combinations of genes whose regional expression patterns best explained the neuroanatomical distribution of depression-related connectivity effects, focusing on the four models with robust generalization to held-out data in Fig. 4b, e, but modeling gene expression in men and women separately using the Brainspan dataset. As above, we found that regional differences in gene expression explained the neuroanatomical distribution of connectivity effects in all four models involving BA25 in men and women, NAc in women, and BA8/9 in men (*r* = 0.64–0.70 for first PLS component, *P* = 0.003–0.023 by permutation testing). Next, we repeated the gene set enrichment analyses, testing for the enrichment of genes that 1) are transcriptionally altered in depression, 2) have been implicated in depression via GWAS or the DisGeNET platform, and 3) exhibit sex-biased brain expression early in life. Despite the fact that the AHBA and Brainspan atlases profiled different brain regions with potentially distinct gene expression patterns, we still found that in all three analyses, a majority of significant enrichments that were observed using the AHBA data replicated in the Brainspan data (Supplementary Figs. S7, 8). We also evaluated the extent to which our AHBA analyses could have been influenced by training a PLS-R model to predict connectivity abnormalities in female brains using predominantly male gene expression data. To test this, we trained PLS-R models to predict the spatial pattern of abnormal connectivity in one sex based on Brainspan transcriptomic data derived from either the same sex, the opposite sex, or both sexes. There was no significant difference in performance across the three conditions (Supplementary Fig. S11), indicating that regional differences in gene expression are more important than sex differences in explaining the spatial distribution of abnormal connectivity in our sample.

**Fig. 5:**
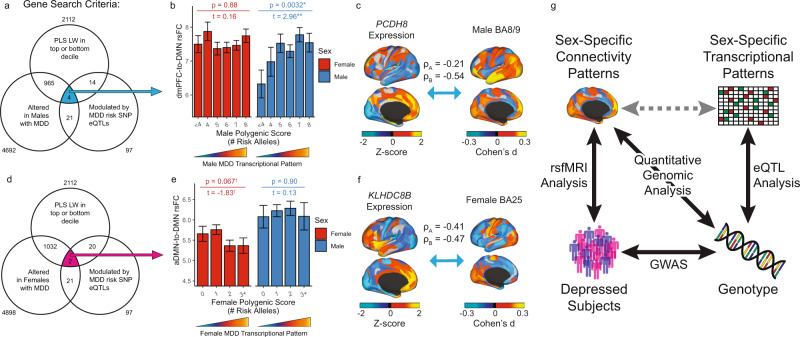
Validating model predictions: inferred expression levels of depression-related genes predicted individual differences in DMN connectivity. **a** Schematic for identifying genes with convergent evidence of abnormal expression in an RNA-seq analysis^8^ (lower left circle), expression regulated by GWAS-confirmed risk variants (lower right circle), and implicated by our male BA8/9 PLS-R model (top circle). Four genes met all three criteria: *PCDH8*, *PRSS16, MRM2*, and *ZKSCAN8P1*. **b** Association between individual differences in DMN-to-dmPFC connectivity in *n* = 1458 UK Biobank subjects (*n* = 537 males) with a history of depression and individual differences in polygenic depression risk at five MDD risk alleles (at SNPs rs72839477, rs67981811, rs12525684, rs2806933, and rs11772627) that have been shown to modulate the expression of the four genes identified in (**a**), i.e. increasing the expression of *PRSS16* and *MRM2* and decreasing the expression of *ZKSCAN8P1* and *PCDH8*^76^, a transcriptional pattern previously associated with depression in men^8^ and demarcated qualitatively in the color bars below the x-axes. As predicted, this association was significant in males (*t* = 2.96, *P* = 0.003, *n* = 537) but not in females (*t* = 0.16, *P* = 0.88, *n* = 921), in a linear model of the effect of genotype on DMN connectivity, covarying for age. Error bars = SEM. **c** *PCDH8* expression (left) was spatially correlated with depression-related connectivity abnormalities seeded from BA8/9 (right) in both AHBA (ρ_A_) and Brainspan (ρ_B_). Qualitatively similar associations were observed for *PRSS16, MRM2*, and *ZKSCAN8P1*. **d** Analogous to (**a**), schematic for selecting genes for validation of the female BA25 model. Two genes met all three criteria: *TMEM161B-AS1* and *KLHDC8B*. **e** Association between individual differences in anterior DMN (aDMN)-to-DMN connectivity in *n* = 1458 UK Biobank subjects with a history of depression and individual differences in polygenic depression risk at two MDD risk alleles (at SNPs rs3099439 and rs7617480) that have been shown to modulate the expression of the two genes identified in (**d**), i.e. increasing the expression of *TMEM161B-AS1* and *KLHDC8B*^76^, a transcriptional pattern previously associated with depression in women^8^ and demarcated qualitatively in the color bars below the x-axes. Individual differences in DMN connectivity were modestly associated with genotype at these two depression risk alleles in females (*t* = –1.83, *P* = 0.067, *n* = 921) but not in males (*t* = 0.13, *P* = 0.90, *n* = 537), in a linear model of the effect of genotype on DMN connectivity, covarying for age. Error bars = SEM. **f** *KLHDC8B* expression (left) was spatially correlated with depression-related connectivity abnormalities seeded from BA25 (right) in both AHBA (ρ_A_) and Brainspan (ρ_B_). Qualitatively similar associations were observed for *TMEM161B-AS1* in AHBA but not in Brainspan. **g** Diagram representing a model in which select GWAS-confirmed genetic risk variants contribute to pathophysiology in depression by regulating the expression of specific genetic pathways that in turn modulate functional connectivity in depression-related brain networks. In this work, we used genotypes at SNPs related to depression via previous GWAS^52^ (bottom right) as instrumental variables to infer associations (grey dotted line) between connectivity patterns in depression (top left) and genes of interest (top right) whose expression in nervous tissue was shown to be modulated by depression-related genotypes in previous eQTL analyses^76^.

Having replicated key findings in the Brainspan dataset, we set out to further validate our PLS results and more directly test the hypothesis that GWAS-confirmed genetic risk variants influence pathophysiology by modulating the brain expression of specific genes, which in turn regulate functional connectivity in depression-related networks. To this end, we aimed to test whether individual differences in the expression of specific depression-related genes were associated with individual differences in functional connectivity in a new dataset, focusing initially on the male BA8/9 PLS-R model, which showed the strongest enrichments for GWAS-related genes (Fig. 4k) and for genes that are transcriptionally altered in men with depression (Fig. 4h). To limit the number of hypotheses tested and reduce the likelihood of false positives, we searched for a convergence of genes meeting three criteria (Fig. 5a): 1) genes with expression patterns that were strongly correlated with abnormal connectivity seeded from BA8/9 in men with depression (i.e. loading weights in the top or bottom decile in the male BA8/9 PLS model in Fig. 4); 2) genes that were transcriptionally altered in the brains of men with depression in a prior report^8^; and 3) genes whose expression is controlled by GWAS-confirmed risk variants for depression (see Online Methods). We identified four genes that met all three criteria: *PRSS16* and *MRM2*, with increased expression in depression, and *ZKSCAN8P1* and *PCDH8*, with decreased expression in depression, regulated by five MDD risk alleles involving rs72839477, rs67981811, rs12525684, rs2806933, and rs11772627 ^76^.

Because datasets including both brain transcriptomic profiling and rsfMRI mapping in human subjects with depression are not available, we instead used an established method to infer relative gene expression levels in the human brain in 1,458 UK Biobank subjects (537 males) with a history of depression, using each subject’s genotype at the five risk alleles specified above to predict the expression of *PRSS16, MRM2, ZKSCAN8P1*, and *PCDH8*^76^. We then tested whether inferred expression levels for these depression-related genes were associated with depression-related rsFC patterns similar to those observed in our cohort of men with depression. As predicted, genotypes involving a larger number of depression risk alleles were associated with progressively increased rsFC between BA8/9 and the DMN in males with a history of depression (Fig. 5b, c). Interestingly, this effect was not present in females with a history of depression (*n* = 921, Fig. 5b), lending further support to a sex-specific role for these genes in modulating connectivity abnormalities in men with depression, as predicted by our PLS-R models.

Third, we performed an analogous analysis in women with depression, focusing on the female BA25 model, which showed the strongest enrichments for GWAS-related genes and for genes that are transcriptionally altered in women with depression. We identified just two genes that met all three criteria described above (Fig. 5d): *KLHDC8B* and *TMEM161B-AS1*, with increased expression in depression, regulated respectively by two GWAS-confirmed risk alleles, rs7617480 and rs3099439. Because the UK Biobank brain parcellation does not include a seed constrained specifically to BA25, we tested whether these SNPs modulate rsFC between UK Biobank’s “anterior DMN” seed, which includes BA25 as well as other anterior DMN areas. As predicted, we found that the depression risk alleles at rs7617480 and rs3099439 were modestly associated with decreased rsFC between the anterior DMN and the rest of the DMN in females with a history of depression, although this trend was not statistically significant (*P* = 0.067, Fig. 5e, f). In contrast, this trend was not present in males with a history of depression (*n* = 537).

Together, these analyses replicate key findings in a separate gene expression atlas and lend independent support to a model linking heritable genetic risk variants, transcriptomic programs, and depression-related connectivity abnormalities (Fig. 5g). Specifically, they are consistent with a model in which select GWAS-confirmed genetic risk variants contribute to pathophysiology in depression by regulating the expression of specific genetic pathways that in turn modulate functional connectivity in the default mode network and other depression-related circuits, acting through sexually divergent mechanisms.

### Simulating implications for biomarker development and future studies

**Fig. 6:**
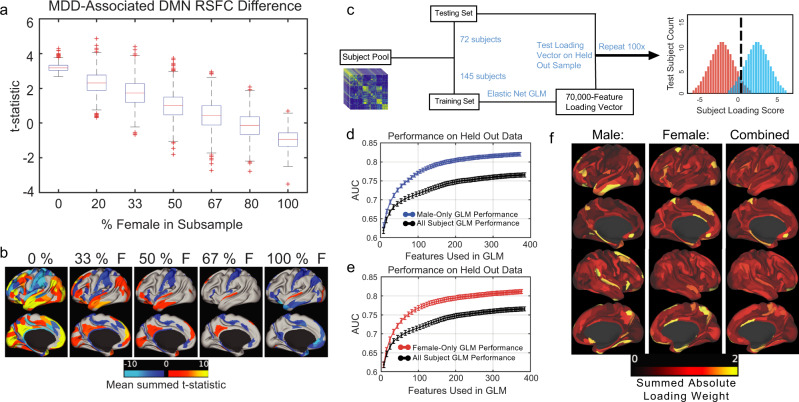
Simulating implications of sex differences for rsfMRI analyses and diagnostic classifier performance. **a** rsFC abnormalities in the DMN vary with the sex composition of the sample. rsFC abnormalities are summarized in terms of the mean t-statistic in mass univariate contrasts of each rsFC within the 77-node DMN, with boxplots depicting the distribution of the mean t-statistic over 1000 bootstrapped subsamples of *n* = 140 MDD subjects and *n* = 70 healthy controls, selected at random without replacement from the full study sample, across seven different sex compositions (x-axis). **b** Color map showing the neuroanatomical distribution of the connectivity abnormalities summarized in (**a**). For each functional parcel, significant t-statistics (*P* < 0.05, FDR *q* < 0.1) were summed across the 77 DMN nodes, averaged across 1000 iterations, and plotted on brain surfaces, with warm colors indicating significant connectivity increases. **c** Schematic for 2:1 hold-out paradigm for training and testing elastic-net regularized general linear models (GLMs) in males only, females only, or in all subjects. **d**, **e** Area under the ROC curve (AUC) for EN-GLMs trained and tested on all subjects (black lines) compared to (**d**) males only (blue line) and to (**e**) females only (red line). **f** Color map showing summed absolute loading weight for all connectivity features related to a given ROI, mapped onto all 360 cortical ROIs, averaged over 100 iterations of each EN-GLM for the male-only models (left), the female-only models (center), and the sex-blind models (right).

The results above define significant sex-specific connectivity abnormalities in the default mode network and to varying degrees, in five transcriptomic regions of interest. To better understand the degree to which sex differences of the magnitude observed here are not only statistically significant but are also strong enough to influence future studies, we performed two concluding analyses. First, motivated by inconsistent findings in rsfMRI studies of depression and varying sample demographics, we tested whether sex differences in rsFC are of sufficient magnitude to significantly alter the results of a study, depending on the sex composition of a study sample, focusing on DMN connectivity abnormalities—one of the most commonly studied targets in depression neuroimaging. To this end, we tested for DMN connectivity differences in 1000 bootstrapped subsamples of our data (*n* = 140 MDD patients, *n* = 70 healthy controls) for each of seven different sex compositions ranging from 100% men to 100% women. We found that the results varied markedly with sex composition (Fig. 6a, b). In general, increased male representation in simulated study sub-samples was associated with more significant hyperconnectivity within the DMN (Fig. 6a). Even samples with 50% women vs. 67% women—a range that is common in existing studies—yielded significantly different results, such that most hyperconnectivity findings were not evident in samples with 67% women (Fig. 6b). These results show that sex differences in rsFC are not only detectable but also biologically significant and could explain some of the variation in rsfMRI results in the previous studies^10,11,13^.

Second, there is substantial interest in developing and optimizing fMRI biomarkers for diagnosing subtypes of depression and other psychiatric conditions and informing treatment decisions^12,17,18,77–79^. To test whether sex differences in rsFC are sufficient to influence the performance of diagnostic fMRI biomarkers, we trained elastic-net regularized general linear models (EN-GLM) to predict depression status based on rsFC (Fig. 6c), trained either on men and women separately or on data from both sexes, iteratively evaluating performance in strictly held-out data (train: 2/3 of sample, test: 1/3 of sample). Compared to an EN-GLM trained and tested on men and women together, independent of sex, sex-specific EN-GLMs showed consistently higher accuracy rates (Fig. 6d, e). To investigate which connectivity features were most consistently predictive of MDD status, the feature-level beta-weights were averaged at each connectivity feature over the 100 GLM iterations, and the absolute values of these connectivity feature beta-weights were then summed across columns for each ROI (Fig. 6f). In accord with our analyses above, the results varied by sex: dorsomedial prefrontal cortex and other regions of the default mode network had consistently high beta weights in the male EN-GLMs, whereas cingulo-opercular regions of the supplementary motor area, superior frontal gyrus, and dorsal anterior cingulate cortex had consistently high beta weights in the female EN-GLMs (Fig. 6f). These results provide further evidence for robust sex differences in rsFC features that consistently predict depression status, and show that the magnitude of these effects is sufficient to influence diagnostic performance.

## Discussion

Female sex is among the strongest known risk factors for depression and a potentially critical contributor to diagnostic heterogeneity, but the underlying neurobiological mechanisms are not well understood. Our results make three contributions to advancing our understanding of those mechanisms. First, they show that depression is associated with distinct connectivity abnormalities in men and women in the default mode network and to varying degrees, in five transcriptomic regions of interest that exhibit sexually dimorphic gene expression changes in depression and are important therapeutic targets. Hyperconnectivity within the default mode network—one of the most commonly studied features of depression—was driven almost entirely by men in two large-scale datasets. Similar to recent observations in RNA sequencing analyses of postmortem brain tissue^8^, sex-specific effects on connectivity were more common than shared effects, and many connections were altered in opposing directions in men and women with depression. Second, our results confirm that sex differences are not only statistically detectable but also of sufficient magnitude that they are likely to be biologically meaningful. rsfMRI analyses of DMN connectivity abnormalities in bootstrapped subsamples with varying sex compositions yielded markedly different results, and diagnostic classifiers trained separately on men and women outperformed those trained on men and women together. Of note, two of the ROIs with the most significant sex-specific effects—dorsomedial prefrontal cortex (BA8/9) and subgenual cingulate cortex (BA25)—are therapeutic targets for deep brain stimulation and TMS^17,18,59^, treatment modalities that are increasingly guided by functional connectivity mapping. Sex is not a known predictor of response to these treatments, but our results suggest that functional targeting strategies could benefit from accounting for sex differences.

Third, our results indicate that a subset of genes that have been implicated in depression may contribute to pathophysiology by modulating functional connectivity in specific circuits. Our data show that regional differences in the expression of partially overlapping but distinct gene sets explain which specific circuits and networks exhibit abnormal connectivity in men and women with depression. The most robust spatial associations were identified for rsFC changes at the BA25 and nucleus accumbens seeds in women, and the BA25 and BA8/9 seeds in men. Consistent with our hypothesis, these gene sets varied by brain region and sex and were enriched for genes linked to GWAS-confirmed risk variants and genes that are known to be differentially expressed in post-mortem RNA-seq studies of depression. Of note, differences between BA25 and BA8/9 in cellular morphology and cortical lamination may also account in part for these gene set differences. We also identified reproducible enrichments for genes with sex-biased expression early in life, and genes involved in synapse function, immune signaling, and neurodevelopment—findings in accord with the results of recent GWAS studies in depression as well as other psychiatric disorders^31,42,80^.

We validated our initial observations supporting this model in three ways. First, we confirmed that depression has sex-specific effects on functional connectivity in two independent, large-scale datasets, replicating key results from the Toronto sample in the UK Biobank database, despite differences in fMRI data acquisition and preprocessing and patient characteristics such as a history of treatment resistance. Second, we confirmed that our PLS results linking abnormal connectivity and gene expression are robust in cross validation and reproducible using two independent gene expression atlases. Third, we were able to formulate predictions about associations between specific GWAS-confirmed risk variants, the expression of specific genes with known transcriptional effects, and individual differences in resting state functional connectivity—and then test them in the UK Biobank dataset, leveraging previously established methods for inferring cortical gene expression. We identified four genes (*PRSS16, MRM2, ZKSCAN8P1*, and *PCDH8*) that were strongly implicated in explaining abnormal dorsomedial prefrontal connectivity in our PLS-R model that are transcriptionally altered in the brains of men with depression, and whose expression is controlled by GWAS-confirmed risk variants. We then tested whether inferred expression levels for these depression-related genes were associated with rsFC patterns similar to those observed in our cohort of men with depression, leveraging the genome sequencing and rsfMRI data from the UK Biobank. In this independent sample, we showed that individual subject genotypes involving a larger number of depression risk alleles—and larger inferred transcriptional abnormalities—were associated with progressively increased rsFC between BA8/9 and the DMN in men with a history of depression, but not in women. Similar trends were observed for a different set of genes implicated in connectivity abnormalities in women with depression, but these effects did not reach significance. Of note, *PCDH8* (Fig. 4c) is an especially promising target for future studies. Its expression in the brain is modulated by a GWAS-confirmed risk variant (rs2806933)^52,76^; its expression is abnormal in the brains of men with depression but not in women with depression^8^; and the neuroanatomical distribution of abnormal dorsomedial prefrontal connectivity in men with depression is correlated with regional differences in the expression of *PCDH8* in both AHBA and Brainspan (Fig. 4c). *PCDH8* encodes a protocadherin integral membrane protein involved in cell adhesion in the brain^81^ in response to neural activity^82,83^. Its expression at synapses has been implicated in long-term potentiation and activity-induced synaptic reorganization^82–84^. Thus, converging evidence from multiple sources indicate that *PCDH8* is well situated to modulate pathophysiology by regulating functional connectivity.

Several important limitations are also noteworthy. First, while our analyses identified a multitude of sex-specific effects, it is also important to emphasize that neither connectivity abnormalities nor their gene expression correlates were totally non-overlapping. On the contrary, and unsurprisingly, there were many effects shared by both sexes. As in other medical conditions in which sex is a risk factor^85–87^, our results suggest that some of the molecular and circuit-level mechanisms contributing to pathophysiology in depression are differentially important in men versus women, while others are shared. Second, rsFC measures are influenced by many factors, and sex differences could be related to a combination of physiological factors like differences in respiration and cardiovascular physiology^88,89^, in addition to changes in synaptic connectivity and other neurobiological mechanisms. Subtle differences in clinical symptom profiles and comorbidities could also contribute: as noted above, some studies suggest that comorbid substance use disorders are more common in men with depression, while comorbid anxiety disorders and atypical symptoms are more common in women with depression^4–7^. Our data also do not speak to the important question of whether the sex differences we observed are due primarily to sex-related biological factors, gender-related psychosocial factors, or a combination of the two. Third, our primary analyses were limited to cross-sectional data acquired at a single point in time from individuals with treatment-resistant depression who were currently experiencing a depressive episode of moderate or severe intensity. Longitudinal studies^90–92^ in more heterogeneous samples will be necessary for determining whether the sex differences we identified are also evident in mild depression, whether they persist in older adults and in women after menopause, and understanding how they vary with the onset, remission, and recurrence of depressive episodes over time. Indeed, the functional connectivity effects that we observed—and sex-related variation in their expression—could be mood state-dependent, attenuating in remission. In accord with this hypothesis, in multisite analyses involving a preponderance of patients in remission, there were no pronounced gender differences in depression effects on brain structure (white matter integrity, cortical thickness)^93,94^. Fourth, our focus on default mode network connectivity was motivated by prior studies of this network, but it is important to emphasize that connectivity abnormalities in the DMN are not specific to depression. Different patterns of DMN dysfunction may play a role in multiple neuropsychiatric disorders, including Alzheimer’s disease, autism, and schizophrenia, as well as depression^38–41^. Multiple GWA studies have identified shared genetic risk factors across numerous neuropsychiatric disorders, consistent with genetic epidemiological data indicating that neuropsychiatric disorders tend to co-occur both within individuals and across individuals in a family^19–23,95,96^. In agreement with GWAS results, fGSEA revealed enrichments in our study not only for depression-related genes but also for genes implicated in schizophrenia, bipolar disorder, and in one model, Alzheimer’s disease (Supplementary Fig. S9). Future studies will be needed to determine the extent to which our findings are specific to depression and whether similar transcriptional and network-level mechanisms may also contribute to other neuropsychiatric conditions.

Finally, gene expression data from the Allen Human Brain Atlas provide powerful tools for understanding the molecular correlates of neuroimaging effects, with proven utility in other contexts^38–41,68^, but they also have limitations. They are derived from brain tissue donated by just five healthy men and one healthy woman, most of whom are middle-aged, so they do not capture the full extent of individual variability in cortical gene expression, and they do not allow us to account sex differences in gene expression in our PLS regression models. To ensure our results were not driven by sex biases in the AHBA dataset, we replicated key findings in a second gene expression atlas (Brainspan) with balanced sex composition. Although the genes explaining abnormal connectivity for each seed in Fig. 4 were enriched for gene sets exhibiting sex-biased expression early in life, we also found that models trained on sex-congruent transcriptomic data did not outperform models trained on transcriptomic data from the opposite sex (Supplementary Fig. S11). This suggests that regional differences in gene expression are more important than sex differences in gene expression in explaining the spatial distribution of abnormal connectivity in our sample, and that the most important genes identified in both the AHBA and Brainspan samples did not show strong differences in expression in men vs. women. Instead, sex-specific effects on connectivity may arise downstream of gene expression programs, e.g. through varying effects on neuronal function in the male vs. female brain. Of course, these findings do not rule out the possibility that future studies incorporating cortical gene expression data from many male and female brains might identify additional gene expression modules with strong sex biases that could not be detected in the present study. Simulations in a recent preprint underscore how these models are also prone to overfitting, especially when the number of observations per feature is relatively small^97^. Mitigating this concern, we showed that the connectivity/gene expression associations we detected are stable and robust in cross-validation in held-out data and generalize to a second gene expression atlas.

Still, PLS regression models are by definition correlative and cannot be used to establish causal relationships between gene expression and connectivity abnormalities. Instead, PLS regression^38–41,68,98^ and related approaches^12,77,99,100^ are powerful, emerging tools in systems neuroscience for inferring complex multivariate associations between connectivity abnormalities and cortical gene expression patterns, and for formulating testable hypotheses about candidate mechanisms, which could be evaluated in future studies. Although we might expect the precise gene loading weights to vary in prospective replication samples^97^, it should still be possible to evaluate inferences about disease-associated gene sets and candidate gene modules. Understanding how gene expression differences in postmortem tissue and GWAS-confirmed risk variants interact to contribute to psychiatric pathophysiology is a fundamental challenge and even formulating hypotheses has been difficult. To this end, our analyses prioritized at least six genes implicated in depression by high-powered GWAS^52^, transcriptional analyses^8^, and our PLS-R models: in females, *TMEM161B-AS1* and *KLHDC8B*, and in males, *PRSS16, MRM2, ZKSCAN8P1*, and *PCDH8—*a known regulator of activity-dependent synaptic reorganization. How these genes and associated biological pathways contribute to depression risk is unknown. Our results provide independent support in two datasets for the testable hypothesis that they could be involved in regulating connectivity changes in depression-related networks.

## Methods

### Subjects

All participants provided informed consent, and research protocols were approved by Institutional Review Boards at all sites. Participants in some studies received a modest cash compensation for their time in participating in the study, as described in the original reports from the respective studies below.

#### Toronto Dataset

This dataset included 384 participants diagnosed with treatment-resistant major depressive disorder and 87 healthy individuals. (As explained below, our primary analysis focused on data from *n* = 371 subjects that met our quality control requirements.) TRD participants were originally recruited from the THREE-D Study (ClinicalTrials.gov ID: NCT01887782), which was a noninferiority trial aimed at assessing the antidepressant efficacy of two forms of non-invasive brain stimulation. To be eligible for inclusion, participants were between 18 and 65 years of age and had no contraindications to MRI, and participants with depression had a MINI-confirmed DSM-5 diagnosis of unipolar depression; a score of ≥18 on the 17-item Hamilton Rating Scale for Depression at their screening visit (HRSD^101^); failed to respond to at least one adequate or two inadequate antidepressant interventions in the current depressive episode; and were on a stable dose of psychotropic medication for at least four weeks prior to the start of the study. Subjects were excluded from the study if they were actively suicidal; had a MINI-confirmed diagnosis of bipolar I or II; a diagnosis of psychotic disorder or psychotic symptoms in the current major depressive episode; or a history of substance dependence or abuse within the past 3 months. All healthy controls had no history of any psychiatric disorder, confirmed by trained assessors in a clinical interview; had a 17-item HRSD score of ≤ 8; and were not currently (or in the last four weeks) on any psychotropic medication, or medication that could significantly affect brain perfusion or activity. For additional details on subject recruitment and a full list of inclusion and exclusion criteria, see^102^.

#### fc1000 Datasets

To supplement our healthy control subject pool, we used publicly available fMRI data from 97 healthy volunteers from the open-source “1000 Functional Connectomes” (fc1000) online resource at (https://www.nitrc.org/frs/?group_id=296). Out of 35 available fc1000 data sets, the “ICBM” (*n* = 86), “NewYork_a” (*n* = 84), and “Cleveland CCF” (*n* = 31) cohorts were selected due to their larger sample size and similar demographic and scanning characteristics to the Toronto dataset (3 T scanner; TR < 3000 ms; scanned with eyes closed). Of these 201 subjects, 31 subjects were excluded because they did not meet our scan quality controls (described below), 25 subjects under age 18 were excluded, and a further 48 subjects under age 38 were excluded to ensure that controls and subjects with depression did not show significant differences in age. To maintain subject confidentiality, all files available through the fc1000 project are completely deidentified and anonymized. Each subject’s age, sex, and handedness data are freely available for download at (https://www.nitrc.org/frs/?group_id=296), with group mean age, sex distribution, and other site- and scanner-related information available at (https://www.nitrc.org/docman/?group_id=296). Note that subjects in this sample were free of psychiatric history by self-report and described as “healthy controls”, but they did not undergo a structured clinical diagnostic interview to confirm this assumption.

#### UK Biobank Dataset

To test the generalizability of our findings involving sex differences in the Toronto MDD cohort, we repeated the analyses in Fig. 2 in an independent subject cohort from the UK BioBank. UK Biobank is a health resource aiming to prevent, diagnose and treat numerous disorders. It is comprised of 502,617 individuals whose genetic and environmental data (e.g. lifestyle, medications) were collected between 2006 and 2010 in the United Kingdom (http://www.ukbiobank.ac.uk/). UKB received ethical approval from the Research Ethics Committee (reference 11: /NW/0382). This study has been approved by the UKB Access Committee (Project #4844). Written informed consent was obtained from all participants. Using data field 20126 (“Bipolar and Major Depression Status”), we identified UK BioBank subjects with completed fMRI scans (https://www.fmrib.ox.ac.uk/ukbiobank/) and classified them as healthy controls (*n* = 1907 males, 1773 females) if they fell under the “No Bipolar or Depression” category, or as “Probable MDD history” (*n* = 537 males, 921 females) if they fell within the “Probable Recurrent Major Depression (severe)”, “Probable Recurrent Major Depression (moderate)”, or “Single Probable Major Depression Episode” categories. Because many patients with a probable MDD history may not have been actively depressed at the time of their scan, our primary analyses in Fig. 2 focused on a subset of subjects in this category who were symptomatic at the time of their fMRI scan, defined as subjects who reported severe MDD symptoms on survey responses taken at the time of their scan. Self-reported symptoms were classified as severe if they responded “nearly every day” to any of the following four UK BioBank data fields: 2050 (frequency of depressed mood in the last 2 weeks), 2060 (frequency of unenthusiasm/disinterest in the last 2 weeks), 2070 (frequency of tenseness/restlessness in the last 2 weeks), or 2080 (frequency of tiredness/lethargy in the last 2 weeks). The age of UK BioBank subjects included in this analysis ranged from 46 to 77, and the means and standard deviations for these groups were as follows: healthy control males (mean = 61.7, sd = 7.6), males with probable MDD history (mean = 60.6, sd = 7.3), males with probable MDD history *and* symptomatic MDD (mean = 58.2, sd = 7.4), healthy control females (mean = 60.5, sd = 7.2), females with probable MDD history (mean = 58.8, sd = 7.2), and females with probable MDD history *and* symptomatic MDD (mean = 56.0, sd = 7.3).

### MRI data acquisition

#### Toronto dataset

Participants with depression (*n* = 384) and healthy controls (*n* = 87) underwent neuroimaging on the same scanner. (As explained below, our primary analysis focused on data from *n* = 371 subjects that met our quality control requirements.) Scan acquisition parameters have been previously published elsewhere^103–105^. Briefly, all scans were acquired on Toronto Western Hospital’s 3 T GE HDx MRI system equipped with an 8-channel phased-array head coil. For each participant, two datasets were acquired: a high resolution, T1-weighted fast spoiled gradient-echo structural dataset (TE = 12 ms, flip angle = 20°, 116 sagittal slices, thickness = 1.5 mm, no gap, 256 ×256 matrix, FOV = 240 mm), and a 10-minute resting-state functional MRI dataset in the eyes closed condition (T2*-weighted EPI, TE = 30 ms, TR = 2000 ms, flip angle = 85°, 32 axial slices, thickness = 5 mm, no gap, 64×64 matrix, FOV = 220 mm).

#### fc1000 dataset

*n* = 84 subjects from the “ICBM” sample *n* = 84 subjects from the “NewYork_a” sample, and *n* = 31 subjects from the “Cleveland CCF” sample were scanned on a 3 T MRI system in the eyes closed condition (TR = 2000, 2000, and 2800 ms respectively; 23, 39, and 31 slices, respectively; 128, 192, and 127 volumes, respectively). The scan durations were 6.4 min, 5.93 min, and 12.81 min (3 scans x 4.27 mins / scan) in the “NewYork_a”, “Cleveland CCF”, and “ICBM” sites, respectively. T1 and T2* MR echo-planar image files for these subjects were downloaded from the “1000 Functional Connectomes” (fc1000) online resource at (https://www.nitrc.org/frs/?group_id=296).

#### UK biobank dataset

*n* = 19,831 subjects from the UK BioBank sample were scanned on one of two 3 T MRI systems in the eyes open condition (TR = 735 ms, TE = 39 ms, 64 slices, slice thickness = 2.40 mm, 490 volumes). Scans lasted for a duration of 6 minutes. Note that these subjects were used for an independent replication analysis in Fig. 2, but their data were not integrated and combined with data from the Toronto and fc1000 datasets. Additional details on fMRI data acquisition parameters can be found at (https://www.fmrib.ox.ac.uk/ukbiobank/)^106^.

### MRI data preprocessing

UK BioBank data were downloaded in a preprocessed format, using a pipeline developed by UK BioBank investigators and described elsewhere^106^. The following preprocessing steps were applied to data in the Toronto and fc1000 samples:

#### Structural MRI data preprocessing

T1 anatomical volumes in the Toronto and fc1000 samples were cropped to a smaller field of view (150 mm in z plane) using FSL’s automated robustfov tool and aligned to the 2 mm MNI atlas template using a rigid, 6-degrees-of-freedom (DOF) FLIRT transformation. This image was segmented into gray matter, cerebrospinal fluid, and white matter masks using FSL’s FAST. A non-linear transformation between the ACPC aligned T1-weighted anatomical image and MNI atlas (2 mm) was estimated using FNIRT. A brain extraction was then performed using a binarized MNI brain mask, transformed from atlas space into native image space using an inverse transformation calculated using FSL’s invwarp tool.

#### Functional MRI data preprocessing

Slice-time correction was performed using FSL’s slicetimer program. Correction for head motion was performed using FSL’s MCFLIRT tool^107^. Functional data were co-registered to the ACPC-aligned T1-weighted anatomical image using FSL’s epi_reg program and transformed into atlas space using the non-linear transformation defined above in the T1 anatomical data. All denoising procedures were performed on these preprocessed, atlas-transformed images.

#### Functional MRI data denoising

Functional data was denoised using the aCompCor strategy^108^ implemented in the CONN toolbox (version 17.0; https://web.conn-toolbox.org). Denoising steps included linear de-trending and nuisance regression (5 principle components from eroded white matter and cerebrospinal fluid masks from the aforementioned tissue segmentation; 6 motion parameters and first-order temporal derivatives; and point-regressors to censor time points with mean frame-wise displacement > 0.2 mm). Residual time-series were band-pass filtered (0.01 Hz < *f* < 0.1 Hz) after regression to avoid reintroduction of nuisance-related variation in the time-series. Finally, temporal masks were created to flag motion-contaminated frames for scrubbing. High-motion volumes were identified by framewise displacement (FD) calculated as the sum of absolute values of the differentials of the three translational motion parameters and three rotational motion parameters. 13 TRD subjects and 33 HC subjects had <5 minutes of scan time after censoring for excessive head motion. These 13 MDD and 33 HC subjects were excluded from further analysis.

#### ComBat harmonization to control for scanner- and site-related effects

Our healthy control subject pool was drawn from four scanner sites: Toronto and three sites in the fc1000 dataset (“ICBM”, “NewYork_a”, and “Cleveland CCF”). To control for scanner- and site-related confounding effects while preserving covariates of interest (in this case: sex, age, and MDD status) in rsFC data, we used the ComBat Harmonization method^109–112^. This method uses an empirical Bayesian framework to estimate additive and multiplicative scanner site effect parameters using parametric empirical priors. The data is then adjusted by subtracting the additive effect parameter and dividing by the multiplicative effect parameter^112^. Effects of depression on rsFC in the DMN in both men and women were similar in an analysis restricted to data from just one site (Supplementary Fig. S2), indicating that COMBAT harmonization was effective for controlling for scanner effects and did not introduce spurious artifacts driving the observed effects in the multi-site sample.

### Data analysis: effects of depression and sex on rsFC in the DMN and five transcriptomic regions of interest (Figs. 1–3)

First, we implemented an unbiased, whole-brain quantification of rsFC abnormalities within the DMN and between the DMN and other functional networks in depression. We restricted this analysis to the DMN and its interaction with other networks, because the DMN is one of the most commonly studied functional networks in depression neuroimaging and restricting our search space would increase statistical power to detect modest sex effects, relative to a fully whole-brain search. Preprocessed BOLD signal time series were extracted for 360 cortical areas defined by an extensively validated functional parcellation^48^ and 19 subcortical regions^49^. These functional parcels were used to generate rsFC matrices (i.e. Fisher-Z transformed correlation matrices) between 77 default mode network ROIs and the rest of the brain. In Fig. 1, we used two-way factorial ANOVA to test for main effects of depression diagnosis and sex-by-depression interactions on features in these rsFC matrices. Post-hoc t-tests were used to identify significantly altered in rsFC features in men with depression vs. healthy control men and in women with depression vs. healthy control women. The Benjamini-Hochberg procedure was used to control the false discovery rate (FDR q < 0.05) ^113^.

In Fig. 2, we replicated our initial findings from the Toronto dataset in an independent sample from the UK BioBank (*n* = 3789 subjects, *n* = 109 of whom had symptomatic MDD), in order to test the extent to which these results generalize to other subject samples scanned and preprocessed under different conditions. This analysis was performed on rsFC matrices represented functional connectivity between six DMN nodes derived from an ICA-based functional parcellation implemented by UK Biobank investigators. That is, data preprocessing, ICA parcellation, and rsFC estimation were and implemented and shared by the UK Biobank, and we downloaded and analyzed these rsFC matrices, which are available for download at https://www.fmrib.ox.ac.uk/ukbiobank/ and included six components: UK Biobank component #5 = posterior cingulate/precuneus (PCC/Precun); component #7 = ventromedial prefrontal cortex (vmPFC); component #9 = medial temporal gyrus (mTG); component #13 = dorsomedial PFC (dmPFC); component #21 = dorsal PFC; and component #49 = inferior parietal lobule (IPL).

In Fig. 3 and Supplementary Fig. S4, we used two-factor ANOVA to test for main effects of depression diagnosis and sex-by-depression interactions on rsFC seeded from each of five transcriptomic regions of interest that exhibited sexually dimorphic gene expression signatures in a recent report^8^: subgenual cingulate cortex (Brodmann Area [BA] BA25), dorsal prefrontal cortex (BA8/9), nucleus accumbens (NAc), orbitofrontal cortex (BA11), and anterior insula. Again, post-hoc t-tests were used to identify significantly altered in rsFC features in men with depression vs. healthy control men and in women with depression vs. healthy control women. Venn diagrams were used to depict rsFC features that were significantly altered in men with depression, women with depression, or both. We also tested whether the number of sex-specific effects (i.e. rsFC features that were altered in men with depression but not women with depression, or vice versa) was larger than expected by chance compared to shuffled data with randomly permuted sex labels.

### Data analysis: PLS regression relating gene expression and abnormal rsFC (Figs. 4, 5)

As described below, we first mapped normative regional gene expression profiles for 58,692 microarray probes covering 21,120 genes in the Allen Human Brain Atlas (AHBA), which provides regional gene expression data from *n* = 6 subjects (*n* = 1 woman, ages 24–57)^46,64,65^, and for normalized microarray expression values for 21,392 genes in the Brainspan dataset^47^, which provides regional gene expression data from *n* = 8 subjects (*n* = 4 women, ages 18–40), to the functional parcellation defined in Fig. 1^48^. Next, we used partial least squares (PLS) regression^66^ to identify genes whose regional expression patterns co-vary with regional depression-related connectivity changes for each of the five transcriptomic seed ROIs listed above: BA25, BA8/9, NAc, BA11, and anterior insula. This analysis was performed separately for depression-related connectivity changes observed in male subjects and in female subjects. To interpret PLS results, we used gene ontology and gene set enrichment analyses to understand which molecular pathways were enriched among the most highly associated genes. Finally, we tested whether disease-associated genetic variants that modulate the expression of the highest-loading genes in nervous tissue exert sex-specific effects on seed-based rsFC to the default mode network in men and women with depression.

#### Mapping regional gene expression to fMRI data

For the Allen Human Brain Atlas analyses, microarray expression data was downloaded for all six human brain donors (H0351.1009, H0351.1016, H0351.1015, H0351.2002, H0351.1012 and H0351.2001) from http://human.brain-map.org/static/download. For the Brainspan analyses, RNA-seq reads per kilobase per million (RPKM) values averaged to genes were downloaded for eight brain donors (H376.X.52, H376.X.53, H376.XI.50, H376.XI.52, H376.XI.53, H376.XI.54, H376.XI.56, H376.XI.60) from https://www.brainspan.org/static/download.html.

Most genes in the AHBA had multiple probes, so probe expression values for the same gene were averaged, as described in previous literature^68,70,114,115^, to obtain gene expression values for 21,120 genes at each microarray sample site. Sample sites (provided in MNI coordinate space) were assigned to all voxels whose Euclidean distance was within a 2 mm radius of the sample site in MNI coordinate space, as recommended in previous literature^114^. If two or more sample sites were assigned to the same voxel, the expression values for a given gene across the multiple sample sites were averaged to yield a representative gene expression value for each gene at that voxel in each subject. These voxel-level gene expression values were then assigned to Glasser parcellation cortical ROIs using grayordinates from parcellated CIFTI files downloaded from the Human Connectome Project (https://www.humanconnectome.org/study/hcp-young-adult). Voxel-level gene expression values were then averaged across all voxels participating in a given Glasser ROI to yield representative gene expression values for 21,120 gene probes at each Glasser ROI. In the interest of modeling normative regional variation in human brain gene expression in the six AHBA subjects, and to avoid the impact of inter-individual variation in gene expression, we used Z-score normalization (i.e. subtracting the mean and dividing by the standard deviation for each regional gene expression profile in a given subject) to normalize regional gene expression data for each of the six AHBA subjects, as recommended in previous literature^38,114^. Because only 2/6 AHBA subjects had microarray data from the right cerebral hemisphere, left and right hemispheric gene expression data in each subject were mirrored as in previous literature^40,116^, such that gene expression values at ROIs with only left hemispheric data were reflected onto their right hemispheric counterparts, and ROIs with right and left hemispheric data in 2/6 AHBA subjects were averaged for each gene. Finally, the subject-normalized representative expression values for each of 21,120 genes at each ROI were averaged across all six brains, creating an aggregate regional gene expression matrix for 348 cortical ROIs that were assigned sample sites in at least one of the six brains (i.e. 360 – 348 = 12 Glasser ROIs were not covered by AHBA gene expression data and were excluded from PLS analyses). This resulted in a 348 ROI-by-21,120 gene matrix, which was then Z-transformed again (i.e. each gene expression vector was demeaned and divided by its standard deviation) to obtain a final matrix of normalized regional gene expression value Z-scores (hereafter referred to as “**X**”).

For Brainspan data, the regional gene expression Z-score matrix was generated similarly for 15 out of 16 regions per hemisphere with available RNA-seq data. These regions included the dorsolateral prefrontal cortex, ventrolateral prefrontal cortex, orbital frontal cortex, anterior cingulate cortex, inferior parietal cortex, superior temporal cortex, inferolateral temporal cortex, primary auditory cortex, primary visual cortex, primary motor cortex, primary somatosensory cortex, amygdala, cerebellar cortex, hippocampus, and striatum. The ‘mediodorsal thalamus’ region was excluded because it was not present in our functional brain parcellation. Glasser HCPMM1 parcellation regions were mapped anatomically to the 15 regions. Genes were excluded if they had zero expression in all regions in donor brain tissue, or if they did not have an associated Entrez ID. Each subject’s regional gene expression vectors were Z-score normalized, reflected across hemispheres, and averaged across male subjects (*n* = 4) and female subjects (*n* = 4), as described above for AHBA data. This resulted in one 30 ROI-by-20,287 matrix representing regional gene expression in females and one 30 ROI-by-20,287 matrix representing regional gene expression in males.

#### Gene-neuroimaging partial least squares regression

Partial least squares regression (PLS-R) was used to uncover latent structures with maximal covariance between regional gene expression and regional connectivity change in MDD. PLS is an established multivariate technique for discovering associations between large numbers of predictor variables and response variables^66^. We trained separate PLS-R models for men and women and for each of five transcriptional regions of interest: BA25, BA8/9, BA11, nucleus accumbens, and anterior insula, resulting in a total of ten models (5 ROIs x 2 sexes) trained on AHBA data. In each model, the predictor matrix was the 348 ×21,120 AHBA regional gene expression matrix “**X**”, described above, and the response variable was a 348-element vector, representing the effect size for depression-related connectivity abnormalities associated with each cortical parcel, seeded from one of the five ROIs. The NIPALS algorithm was used to approximate PLS-R results using published code^69^, which is available at (https://github.com/jmmonteiro/spls). This resulted in PLS components for each seed ROI representing sex-specific latent structures of covariance between gene expression and MDD-associated connectivity change (Fig. 4a). Stability of PLS loading weights for individual genes in the “**X**” predictor matrix was estimated using a bootstrap procedure, in accordance with previous literature^68^: the 348 ROIs (rows) of both the “**X**” and “**Y**” matrices were identically resampled with replacement and run through PLS over 10,000 resampling iterations to obtain standard deviations for loading weights of each gene (column) in the “**X**” matrix. For each PLS model, the ratio of each gene’s empirical PLS loading weight to its loading weight standard deviation from the bootstrap procedure was used to rank the genes. All normalized gene LWs for each of the 10 PLS models can be found in Supplementary Table 2. For sex and seed-region pairs that yielded significant and reproducible PLS models using AHBA data (Fig. 4b–e), additional PLS models were trained in the same manner using female- and male-specific regional gene expression matrices derived from Brainspan data (described above) to predict depression-related connectivity changes observed in females and males, respectively.

#### Statistical testing of PLS components

To test whether the observed gene-neuroimaging relationships uncovered by the PLS models were statistically significant, we compared the empirical Pearson correlation between PLS components derived using real data to null distributions of 10,000 Pearson correlations derived using 10,000 spatially permuted response vectors, **“Y**_**k**_”, for k = 1:10,000. In this null model (“spin test”), the rows of the neuroimaging vector “**Y**” were permuted using random rotations of the spherical projection of the cortical surface, performed using a published spatial permutation technique designed to preserve the native correlational structure of variables across the cortical surface^117^. This was accomplished using the ‘rotate-parcellation’ package by Frantisek Vasa, which is available for download at https://github.com/frantisekvasa/rotate_parcellation. By preserving local auto-correlations in cortical surface variables, the “spin test” provides a null model with more stringent controls for false positives, compared to a random permutation null model. For comparison with previous work, we also generated a less stringent, spatially naïve null model by randomly permuting all 348 ROIs in the **“Y”** vector over k = 10,000 iterations. For a given iteration, the Pearson correlation between the gene expression scores (i.e. the projection of the gene expression predictor matrix into its computed loading weights **“u**_**k**_**”**) and the permuted connectivity abnormality vector **“Y**_**k**_” was used as a test statistic (Eq. (1)):

1 $${\rho }_{k}={{{{{\rm{Corr}}}}}}({{{{{\bf{X}}}}}}\;\ast \;{{{{{{\bf{u}}}}}}}_{{{{{{\bf{k}}}}}}},{{{{{{\bf{Y}}}}}}}_{{{{{{\bf{k}}}}}}})$$

where for iteration k of 10,000 iterations, the test statistic is the correlation *ρ*_*k*_, **X** is the gene expression predictor matrix, **u**_**k**_ are the null gene loading weights computed by PLS on iteration k, and **Y**_**k**_ is the permuted “**Y**” seed- and sex-specific connectivity change vector for iteration k. Ten separate null models were computed: one for each of five seed-ROI response vectors “**Y**” in males and one for each seed-ROI response vector in females. We then compared the distributions of null correlations *ρ*_*k*_ in each seed-ROI specific null model in each sex to the empirical correlations *ρ*_*m*_ (Supplementary Fig. S5) observed between the connectivity change response variables and gene expression scores (i.e. projection of **X** into the respective model loading weights **u**_**m**_) in the PLS models derived from unpermuted data, using the same computation as for the null correlations (Eq. (2)):

2 $${\rho }_{m}={{{{{\rm{Corr}}}}}}({{{{{\bf{X}}}}}}\;\ast \;{{{{{{\bf{u}}}}}}}_{{{{{{\bf{m}}}}}}},{{{{{\bf{Y}}}}}})$$

We defined significant PLS components to be above the 95^th^ percentile of the null distribution of correlations *ρ*_*k*_ for the spatially rotated (p_spin_) and randomly permuted (p_rand_) null models described above. All p-values were adjusted for multiple comparisons (10 PLS-R models tested: 5 ROIs x 2 sexes) using the Benjamini-Hochberg approach for FDR correction^118^.

#### Reproducibility testing of PLS components

To test whether the observed gene-neuroimaging relationships uncovered by the PLS models were reproducible, we performed a ten-times ten-fold cross validation of PLS models in held-out data. Separate male and female PLS models were trained as before but using random 90% sub-samples of the male and female subject pools, with equal proportions of subjects with depression in the 90% training and 10% testing sub-samples. PLS models trained on 90% of subjects were then tested on their ability to predict depression-related effects on rsFC in the remaining 10% held-out subjects. Predictive ability in held-out data was measured using the same Pearson rho test statistic as above, but with loading weights (**u**_**train**_) trained on 90% of data and response vectors (**Y**_**test**_) derived from 10% held out subject sub-samples:

3 $${\rho }_{ho}={{{{{\rm{Corr}}}}}}({{{{{\bf{X}}}}}} \;\ast \; {{{{{{\bf{u}}}}}}}_{{{{{{\bf{t}}}}}}{{{{{\bf{r}}}}}}{{{{{\bf{a}}}}}}{{{{{\bf{i}}}}}}{{{{{\bf{n}}}}}}},{{{{{{\bf{Y}}}}}}}_{{{{{{\bf{t}}}}}}{{{{{\bf{e}}}}}}{{{{{\bf{s}}}}}}{{{{{\bf{t}}}}}}})$$

Where *ρ*_*ho*_ is the Pearson rho test-statistic in held-out data and X is the 21,120-gene expression predictor matrix described above. This was calculated 100 times (ten times 10-fold cross validation) using 100 different random sub-samples of 10% held out subjects for each PLS model. The depression diagnostic labels were then randomly permuted and the null *ρ*_*ho*_ was computed across 100 iterations (ten times ten-fold cross validation) again for shuffled data. For each iteration of 10-fold cross-validation, the mean Pearson rho across 10 folds from real and permuted data was compared using a corrected resampled t-test^119,120^. This generated 10 p-values, each representing a different random 10-fold split of the dataset. To determine statistical significance, we Bonferroni-corrected the 10 p-values and then applied an omnibus hypothesis test to determine whether gene-rsFC associations in training data were reproducible in any train-test splits of our dataset, as described in previous neuroimaging work^69,121,122^.

#### Bioinformatic analysis of PLS results

For statistically significant PLS models, the resulting ranked gene lists were tested for over-representation of gene ontology (GO) terms (Supplementary Fig. S10) and for gene sets of interest (listed in Supplementary Table 3) using the online GOrilla tool (http://cbl-gorilla.cs.technion.ac.il/), and the Bioconductor “fGSEA” package (https://bioconductor.org/packages/release/bioc/html/fgsea.html)^123^, respectively. fGSEA analyses were performed using 10,000 null permutations and all other settings set to defaults. The org.Hs.eg.db package, version 3.11.4, which freely available for download in R through Bioconductor, was used to interconvert gene identifiers between entrez ID’s, Ensembl ID’s, and HGNC symbols.

#### PLS result validation using genotype data

To determine whether brain expression of genes implicated in our PLS models predict individual differences in rsFC in a new dataset, we leveraged the Genome Tissue Expression Database (https://www.gtexportal.org/home/datasets; significant cis-QTL variant-gene pairs v8) to identify SNPs modulating nervous system tissue expression levels (i.e. ‘expression quantitative trait loci’, or ‘eQTLs’) of sex-specific genes implicated by our PLS models. We then tested whether different alleles of these eQTLs exerted an additive effect on rsFC in 1458 UK Biobank subjects with a probable history of MDD (see UK Biobank subjects description above) who were genotyped and rsfMRI-scanned, testing males (*n* = 537) and females (*n* = 921) separately. To limit the number of SNP-rsFC associations tested, we restricted our eQTL search to SNPs that have previously shown a genome-wide significant association with depression in two separate cohorts from the most recent large-scale GWAS^52^, and to genes that meet the following conditions:

1. PLS loading weight in the top or bottom decile among 21,120 gene loading weights from each of four significant PLS models of depression-related rsFC changes in female BA25, female NAc, male BA25, and male BA8/9.
2. Expression in nervous system tissues modulated in the same direction by MDD GWAS SNPs from the most recent GWAS^52^ and by depression status in post-mortem transcriptional studies^8^.

Tested SNPs were also controlled for standard QC measures: they were excluded if they showed missingness in >10% of subjects, minor allele frequency >5%, and significant deviation from Hardy-Weinberg equilibrium (determined using Chi-square test with alpha=0.05 and 1 degrees of freedom). Subjects were excluded if their sex recorded in the dataset (UKB data field 31) was not identical to their genotype-inferred sex (UKB data field 22001) or if they were determined by UK Biobank protocols^124,125^ to be outliers based on heterozygosity and SNP missingness (UKB data field 22027).

This search yielded two eQTLs that promoted female-specific transcriptional patterns in depression, and five eQTLs that promoted male-specific transcriptional patterns in depression: In females, the MDD risk allele of the SNP rs7617480 increased nervous tissue expression of KLHDC8B, and the MDD risk allele of rs3099439 increased expression of TMEM161B-AS1^76^. In males, the MDD risk alleles of rs72839477 and rs12525684 increase nervous tissue expression of PRSS16, the MDD risk allele of rs11772627 increases expression of MRM2 (alternatively known as ‘FTSJ2’ or ‘FJH1’), the MDD risk alleles of rs72839477 and rs67981811 decrease expression of ZKSCAN8P1 (alternatively known as ‘ZNF192P1’ or ‘ZNF389’), and the MDD risk allele of rs2806933 decreases expression of PCDH8^76^. We then sought to determine whether these MDD risk alleles that promote sex-specific transcriptional patterns also promote sex-specific connectivity patterns in depression at the seed regions tested. In the analysis in Fig. 5a–c, PRSS16, MRM2, ZKSCAN8P1, and PCDH8, were spatially associated with male-specific connectivity changes in depression at the BA8/9 seed in our PLS-R models in Fig. 4, so we tested whether connectivity between the BA8/9 seed (i.e. UK Biobank ICA node 21) and the rest of the DMN (i.e. UK Biobank ICA nodes 5, 7, 9, 13, and 49) was additively associated with the number of MDD risk alleles across the five SNPs listed above. Likewise, in the analysis in Fig. 5d–f, KLHDC8B and TMEM161B-AS1 were spatially associated with female-specific connectivity changes in depression at the BA25 seed in our PLS-R models in Fig. 4, so we tested whether connectivity between BA25 (approximated in the UK Biobank parcellation as the “anterior DMN” parcel, i.e. UK Biobank ICA node 7) and the rest of the DMN (i.e. UK Biobank ICA nodes 5, 9, 13, 21, and 49) was additively associated with the number of MDD risk alleles across the two SNPs listed above. To investigate these SNP-rsFC relationships in the context of depression pathophysiology, we tested for linear associations between MDD risk allele count and DMN rsFC in 1458 UK Biobank subjects (921 females) with a ‘Probable MDD history’ (see UK Biobank subjects description above). Quantitative trait modeling of mean rsFC across the DMN nodes listed above was performed using a linear model (R function ‘lm’) with genotype (represented as an integer sum of MDD risk alleles in a given subject across the MDD GWAS SNPs above) as the predictor variable, and age as a covariate. Imputed genotype data (UKB Field 22828) was downloaded from UK Biobank for all 19,831 rsfMRI-scanned subjects using the ‘gfetch’ tool (https://biobank.ctsu.ox.ac.uk/showcase/showcase/docs/instruct_gfetch.html). Subject genotype data for the SNPs listed above were extracted from the downloaded.bgen files and converted to binary PED format using qctool version 2 (https://www.well.ox.ac.uk/~gav/qctool_v2/) for quantitative trait association analysis in R. Multiple comparison correction was not performed due the small number of hypothesis-driven multi-allelic score effects (*n* = 2) that were tested.

### Data analysis: simulating the impact of sex differences in rsFC (Fig. 6)

To better understand the degree to which sex differences of the magnitude observed here could influence future studies and biomarker development efforts, we performed two concluding analyses. First, to determine whether sex-specific effects were large enough to influence study outcomes based on sex composition, we simulated 1000 MDD rsFC studies using sub-samples with varying sex compositions, ranging from 0% female to 100% female. In each simulated study, 140 MDD subjects and 70 HC subjects were sub-sampled from our original cohort of 553 ComBat-harmonized subjects using one of seven different sex-compositions (0% female, 20% female, 33% female, 50% female, 67% female, 80% female, and 100% female) applied equivalently to the MDD and HC sub-samples. For each sub-sample iteration, two-tailed t-tests were used to determine significance of MDD effects at each DMN rsFC feature, as well as at the mean of all rsFC features connecting the 77 DMN nodes. As above, the Benjamini-Hochberg procedure was used to control the false discovery rate (FDR q < 0.05) ^113^.

Second, we trained elastic-net regularized general linear models (EN-GLMs) in a sex-specific manner to predict MDD status in separate hold-out samples of males and of females, and we compared these sex-specific EN-GLMs to an EN-GLM trained and tested on samples of combined males and females. 145 subjects were randomly selected from each grouping (male, female, or combined sex) to train the respective EN-GLM, and a separate and non-overlapping group of 72 subjects was randomly selected from the same grouping to test the sensitivity and specificity of each trained EN-GLM (Fig. 6c). This procedure was performed 100 times to obtain a mean performance level (i.e. area under the ROC curve for predicting MDD status in the held-out sample) for the combined-subject, male-only, and female-only EN-GLMs (Fig. 6d, e). Data from the 72 test subjects were strictly held out from all aspects of the training procedure on each iteration. In each EN-GLM, an alpha value of 0.5 was used to allow for equal parts ridge and lasso regularization. The GLM was performed with the MATLAB function “lassoglm”, which iterated over 80 different lambda values, allowing for comparison of GLM performance at different numbers of features (ranging from 1 to ~400) included in the GLM. The feature-level beta-weights were averaged over the 100 GLM-iterations to yield robust beta-weights at each connectivity feature. The absolute value of these connectivity feature beta-weights was then summed across columns for each ROI, yielding a summed absolute loading weight for each of the 379 ROIs in our analysis. The summed absolute loading weight was then plotted on a brain surface (Fig. 6f) to provide an anatomical depiction of features relevant to MDD diagnosis prediction for each of the three EN-GLMs.

### Reporting summary

Further information on research design is available in the Nature Research Reporting Summary linked to this article.

## Supplementary information

Supplementary Information

Reporting Summary

## Data Availability

There were no new raw data generated in this study. All preprocessing and analysis were performed on pre-existing data sets as described in the Methods section. The THREE-D study fMRI and UK Biobank data are available under restricted access to protect patient privacy. Access can be obtained upon reasonable request by contacting the authors of this paper who manage each dataset: Dr. Jonathan Downar for the Three-D data set, and Dr. Andrew Macintosh for the UK Biobank dataset. UK Biobank access can also be obtained via a centralized application process at www.ukbiobank.ac.uk/enable-your-research/apply-for-access. The Allen Human Brain Atlas and Brainspan datasets are freely available and may be downloaded at the links provided in the Methods section. The gene ranking results of partial least squares regression analyses applied to AHBA and Brainspan data are made available in Supplementary Table 2, and Gene Ontology terms derived from those ranked gene lists are made available in Supplementary Table 3.

## Code availability

The code used for machine learning analyses described in this manuscript is made available along with example data in the following Github repository: https://github.com/AlexTalishinsky/Sex_Dimorphic_Depression_rsFC_Gene_Correlates. The original version used for the manuscript is citable via the Zenodo repository at the following link: 10.5281/zenodo.6825246. No novel algorithms were used in the analyses therein.
