# Supplementary material for: Regional gene expression signatures are associated with sex-specific functional connectivity changes in depression
Source: Nat Commun. 2022 Sep 28;13:5692. doi: 10.1038/s41467-022-32617-1 (PMC9519925; doi:10.1038/s41467-022-32617-1)
Supplement: Supplementary file 1 — Supplementary Information [file 41467_2022_32617_MOESM1_ESM.pdf]

Regional gene expression signatures are associated with sex-specific functional connectivity changes in depression

Aleksandr Talishinsky, Jonathan Downar, Petra E. Vértes, Jakob Seidlitz, Katharine Dunlop, Charles J. Lynch, Heather Whalley, Andrew McIntosh, Fidel Vila-Rodriguez, Zafiris J. Daskalakis, Daniel M. Blumberger, Conor Liston

Supplementary Material

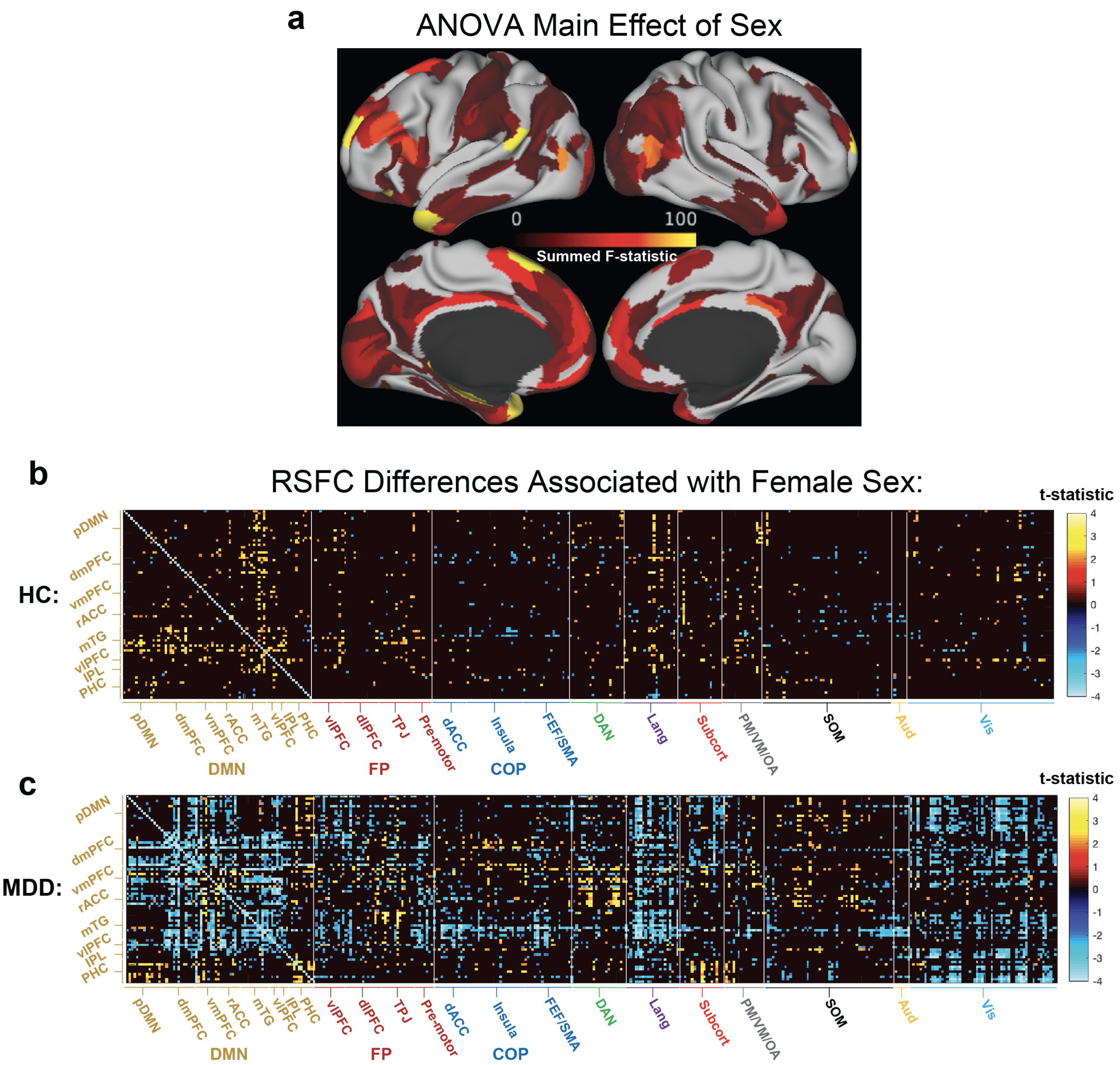

Supplementary Figure 1. ANOVA Main Effect of Sex. a. Summed one-way ANOVA F-statistics representing main effect of sex on rsFC with 77 default mode network (DMN) ROIs for each of 360 HCPMM1 ROIs plotted on a brain surface. All effects plotted are significant with FDR q<0.05. **b.** Significant (unadj. p<0.05) t-statistics representing rsFC effects associated with female sex in healthy subjects. **c.** Significant (unadj. p<0.05) t-statistics representing rsFC effects associated with female sex in subjects with MDD.

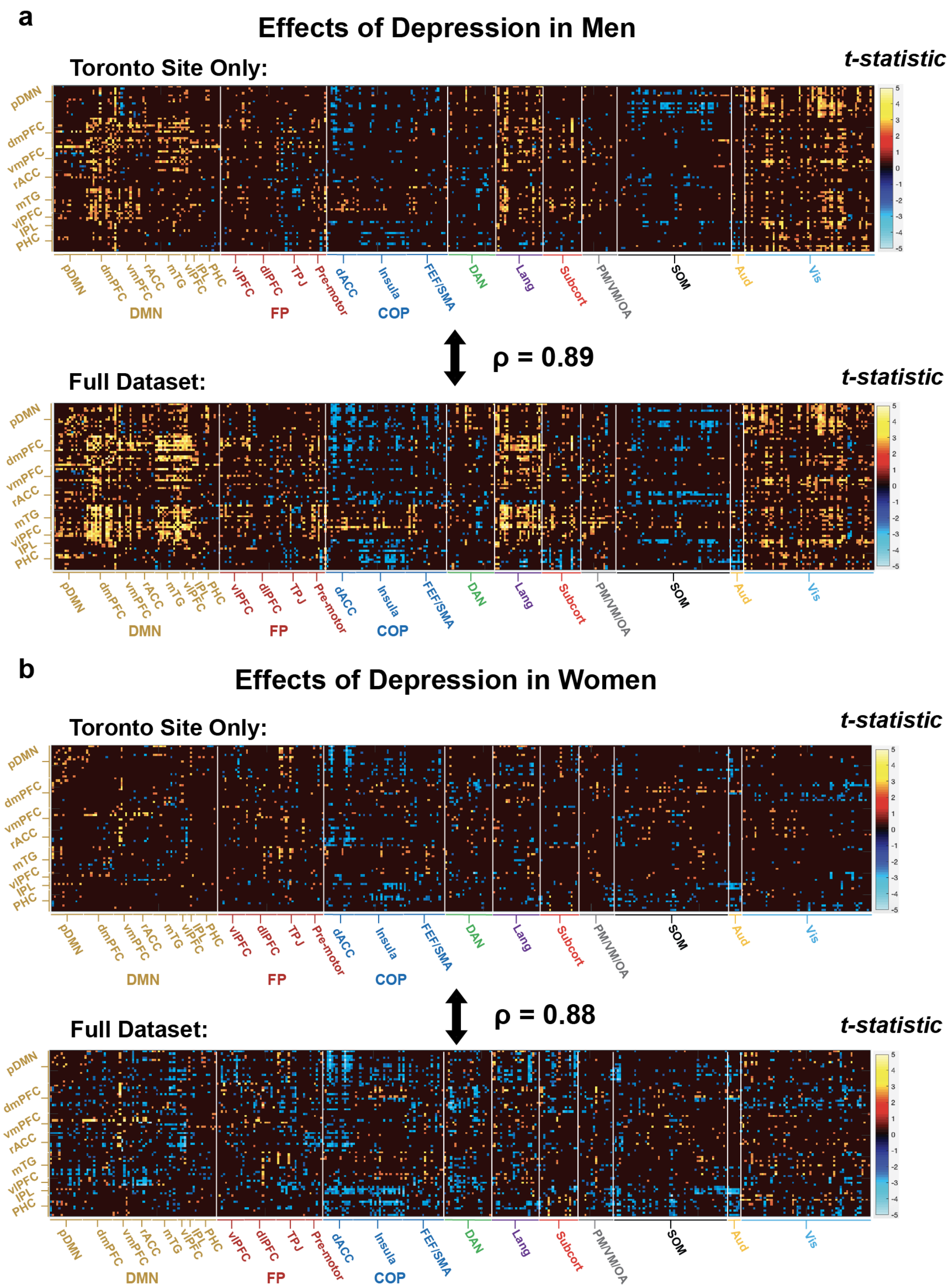

Supplementary Figure 2. Two-tailed t-test Results Pre- and Post- ComBat Harmonization. To ensure that the ComBat Harmonization technique was not introducing significant artifactual effects in our analysis, we compared the results of **(a)** male and **(b)** female MDD vs healthy control t-tests at each of 26,180 unique connectivity features between 77 DMN nodes (in rows) and 360 HCPMM1 and 19 freesurfer subcortical regions (in columns) before ComBat harmonization (top panels) using only subjects from the Toronto dataset (n=148 male MDD, n=33 male HC, n=223 female MDD, n=52 female HC) who were scanned in the same scanner with the same protocol, and after ComBat Harmonization using 553 subjects from two sites. Pre- and post- ComBat harmonization t-statistics were correlated over 26,180 connectivity features in each sex, resulting in Pearson correlations of 0.89 for males, and 0.88 for females, indicating a high degree of concordance in the direction of MDD-related effects. Qualitatively, sex-specific results highlighted in this paper, including male-specific DMN hyperconnectivity, are present regardless of ComBat harmonization.

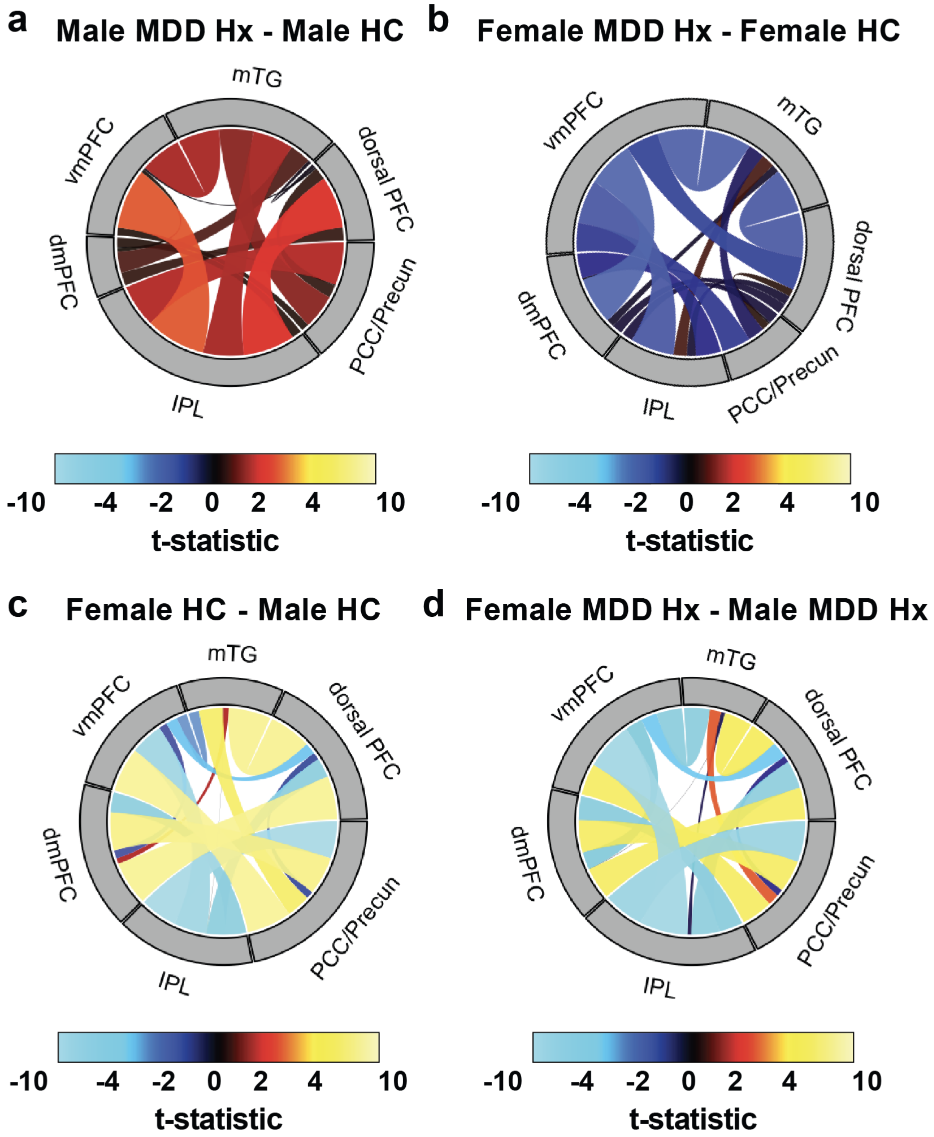

Supplementary Figure 3. Effects of MDD history and sex on DMN rsFC in UK Biobank subjects. Plots depicting rsFC between ICA-nodes restricted to DMN regions, described in Fig. 2f, in four groups of UK BioBank subjects: Male MDD Hx, males with a probable history of MDD (n=537); Male HC, healthy males (n=1907); female MDD Hx, females with a probable history of MDD (n=921); female HC, healthy females (n=1773). Warm colors denote increased connectivity in subjects with probable MDD history compared to controls **(a-b)** or in women compared to men **(c-d)**.

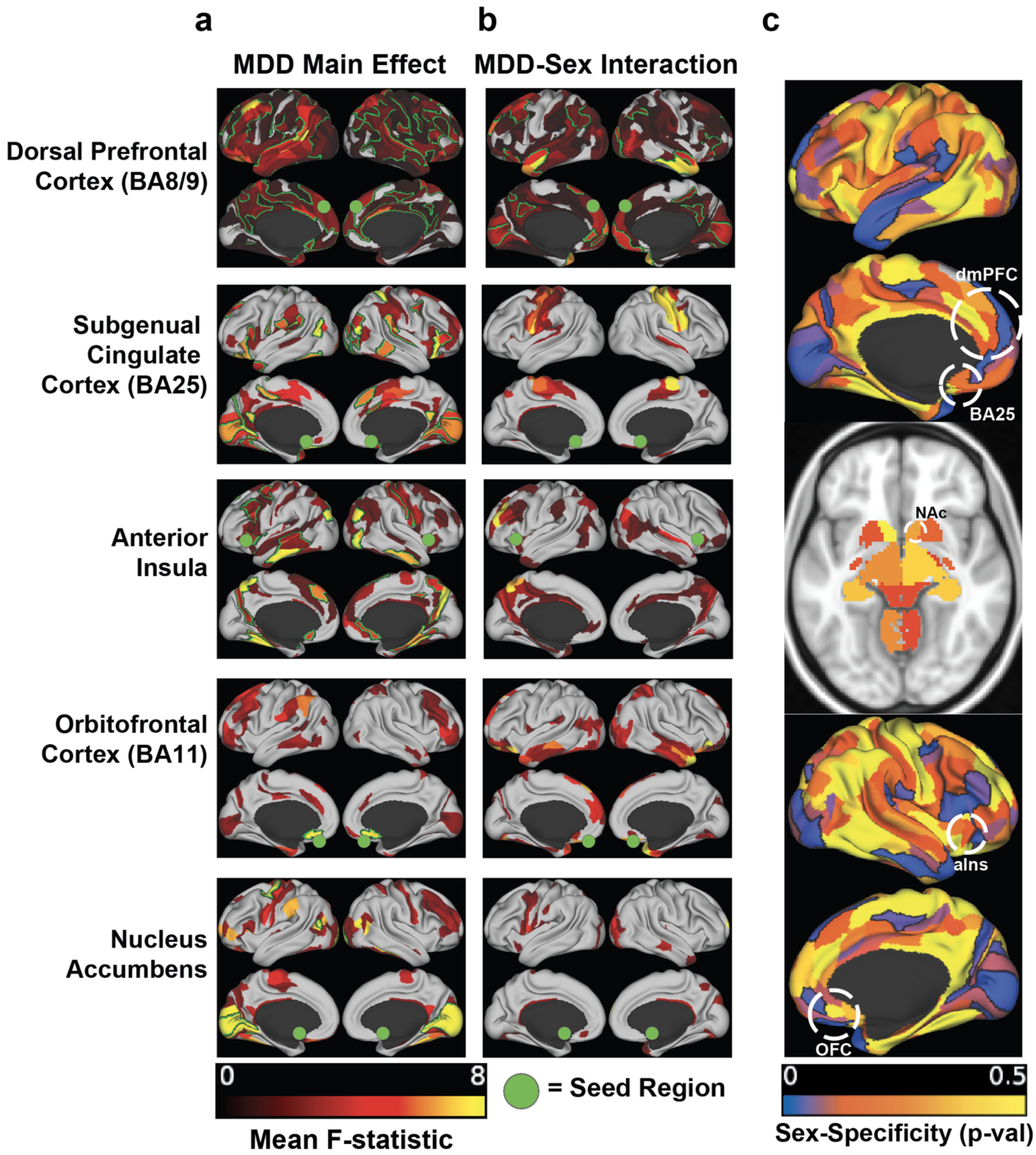

Supplementary Figure 4. Sex-specific rsFC effects at five transcriptionally-defined ROIs. a-b. For a given seed region (green circles, labeled in rows), two-way ANOVA (factors = MDD status, sex, n=553) was performed at each rsFC feature associated with an brain parcel within that seed region, and F-statistics representing significant ($p<0.05$) **(a.)** main effects of MDD and **(b.)** MDD-sex interaction effects were meaned over brain parcels participating in each seed region and plotted on a brain surface. Regions with FDR $q<0.05$ effects are outlined in green. **c.** Brain spatial map of p-values representing the percentile of the correlation between male and female depression effects on rsFC (t-stat via two-tailed t-test) in a null distribution of 1,000 correlations of depression effects on rsFC between two randomly assigned sub-groups of subjects (i.e. not aligned with sex). Male-female correlations below the fifth percentile ($p<0.05$) are highlighted by black borders.

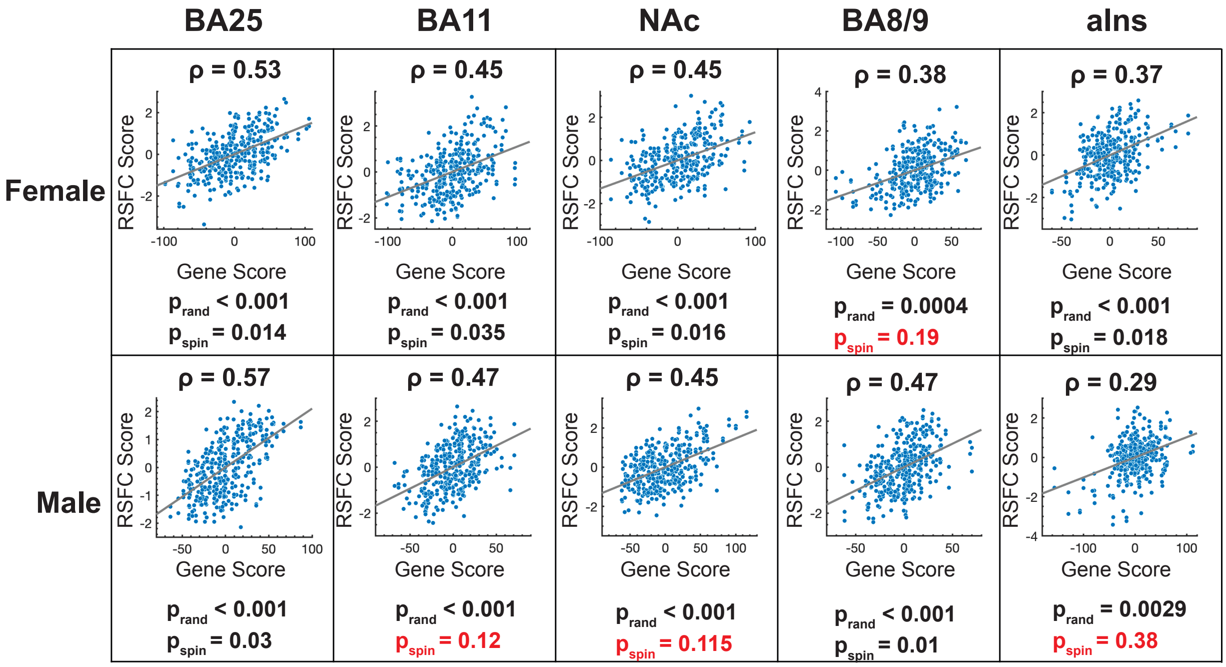

Supplementary Figure 5. Gene-Neuroimaging Partial Least Squares Regression Score Correlations. For 10 PLS-R models at five seed regions (columns) in females (top panels) and in males (bottom panels), Pearson correlation coefficients between PLS-R component loading scores are shown above scatter-plots indicating how well PLS-R Gene component scores (x-axes) can predict MDD effects on rsFC (y-axes). Each dot in the scatter plot represents a cortical region in the HCPMM1 parcellation. Significance was assessed for each of the 10 PLS-R models using both a random spatial permutation test and a random spatial rotation "spin" test (described in Methods). Associated p-values (p_{rand} and p_{spin}, respectively) are plotted below each scatter plot, with non-significant p-values in red. All p-values are adjusted for multiple comparisons using the Benjamini-Hochberg FDR correction approach (see Methods).

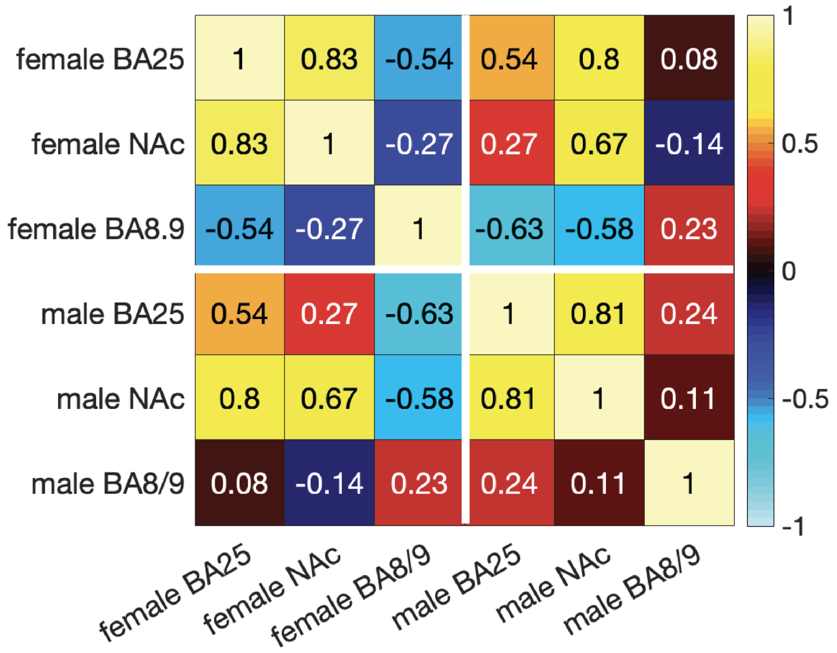

Supplementary Figure 6. Spearman correlation of loading weights across 21,120 genes in six PLS models from (Fig. 4f-g).

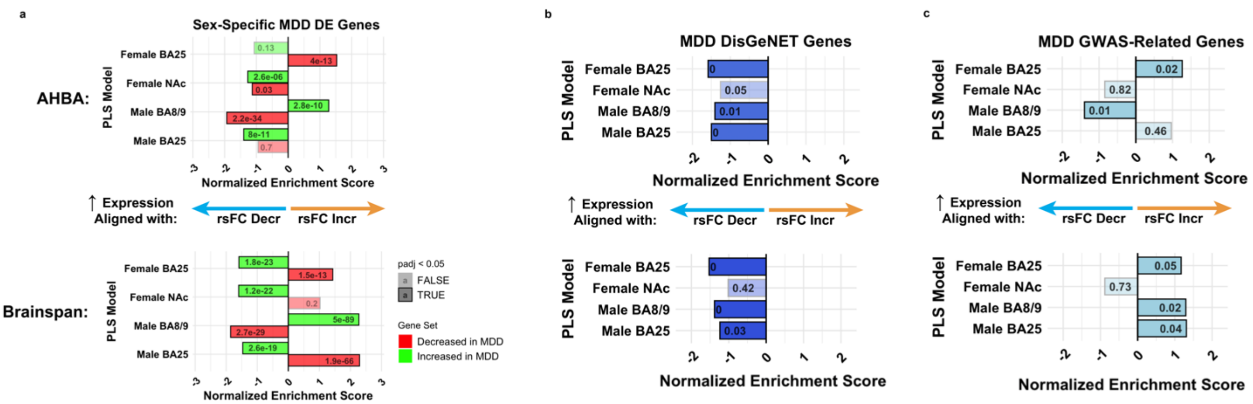

Supplementary Figure 7. PLS-R Models trained on AHBA and Brainspan gene expression data show convergent results on enrichment of depression-related gene sets. a. fGSEA results for differentially expressed genes in depression using loading weight-ranked gene lists derived from PLS analysis with gene predictors derived from AHBA (top) and Brainspan (bottom) datasets. In all four PLS models shown, genes predicting the spatial distribution of connectivity abnormalities in MDD are enriched for genes that show increased (green) or decreased (red) expression in brain tissue donated by MDD subjects of the corresponding sex. fGSEA-generated normalized enrichment scores (x-axis), p-values (plotted in each bar), and adjusted p-values (darkened bar color if FDR q<0.05) are plotted. Negative enrichment scores denote enrichment among genes with negative LWs in the PLS regression model, and positive enrichment scores denote enrichment among genes with positive LWs. **b.** fGSEA results for depression-related risk genes as defined in the DisGeNet database using loading weight-ranked gene lists derived from PLS analysis in AHBA (top) and Brainspan (bottom) datasets. **c.** fGSEA results for genes whose nervous tissue expression is modulated by significant SNPs from the most recent large-scale GWAS for depression using loading weight-ranked gene lists derived from PLS analysis in AHBA (top) and Brainspan (bottom) datasets. Full listing of genes in all gene sets can be found in Supplementary Table 3.

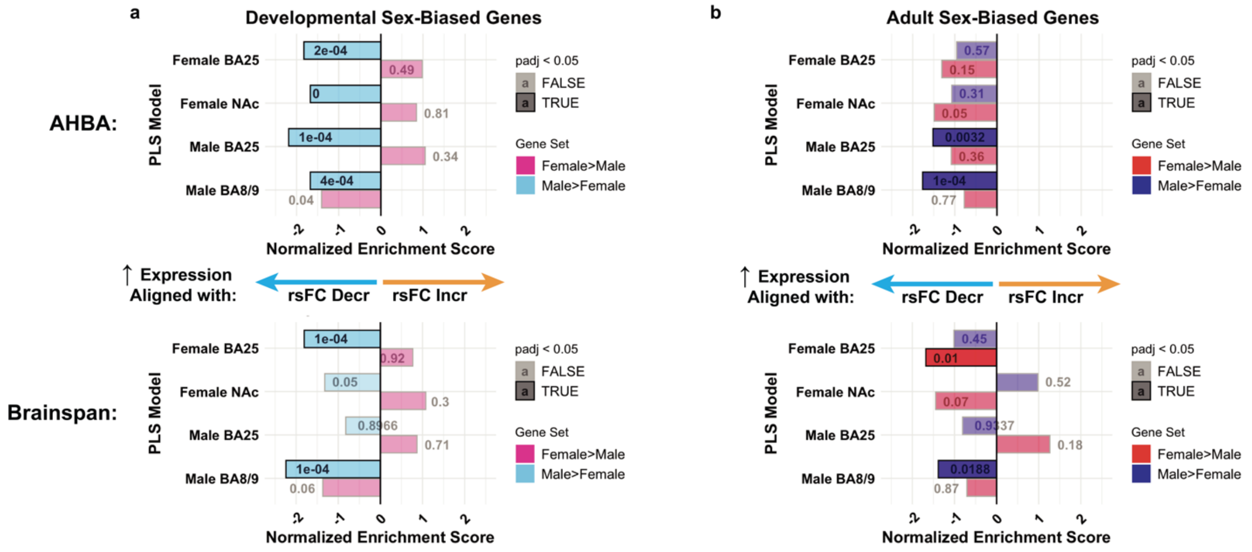

Supplementary Figure 8. PLS-R Models trained on AHBA and Brainspan gene expression data show convergent results on enrichment of sex-biased gene sets. a. fGSEA results for genes differentially expressed between males and females during development[1] (i.e. 4 post-conception weeks – 20 years old) using loading weight-ranked gene lists derived from PLS analysis with gene predictors derived from AHBA (top) and Brainspan (bottom) datasets. In all four PLS models shown, genes predicting the spatial distribution of connectivity abnormalities in MDD are enriched for genes that show decreased (light blue) expression in brain tissue donated by female subjects in one or both datasets. fGSEA-generated normalized enrichment scores (x-axis), p-values (plotted in each bar), and adjusted p-values (darkened bar color if FDR q<0.05) are plotted. Negative enrichment scores denote enrichment among genes with negative LWs in the PLS regression model, and positive enrichment scores denote enrichment among genes with positive LWs. **b.** fGSEA results for genes differentially expressed between males and females during adulthood[2] (i.e. >20 years old) using loading weight-ranked gene lists derived from PLS analysis in AHBA (top) and Brainspan (bottom) datasets. Full listing of genes in all gene sets can be found in Supplementary Table 3.

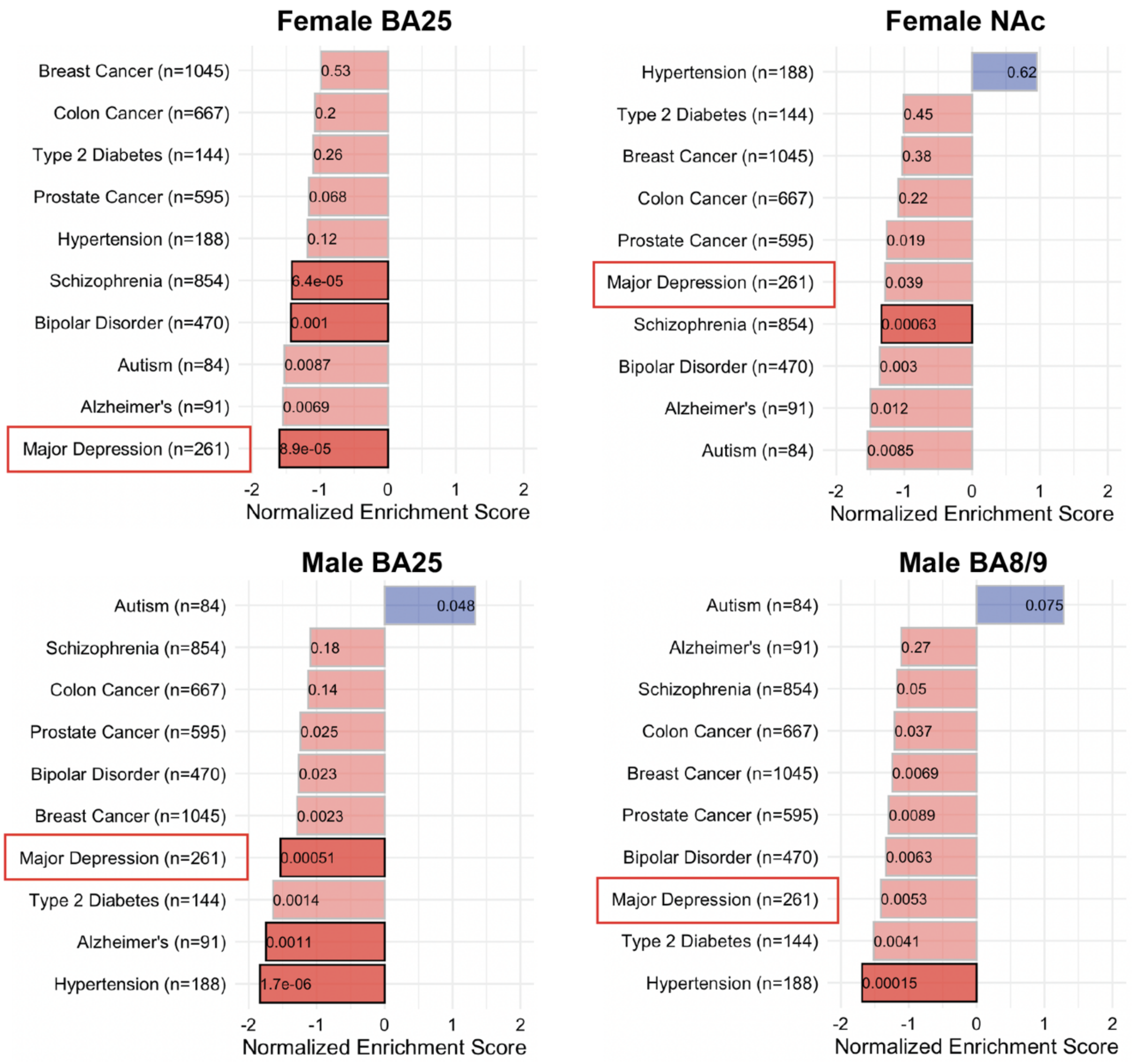

Supplementary Figure 9. PLS-R Models trained on AHBA gene expression data show enrichment of gene sets related to depression, neuropsychiatric disorders, and hypertension. fGSEA results for disease-related genes for 10 diseases (in rows of each panel) defined in the DisGeNet database using loading weight-ranked gene lists derived from PLS models trained on AHBA gene expression data to predict depression-related connectivity changes in females at the BA25 seed (top left), in females at the NAc seed (top right), in males at the BA25 seed (bottom left), in males at the BA8/9 seed (bottom right). Horizontal bars depict fGSEA normalized enrichment scores for each gene set in loading-weight-ranked gene lists from each PLS model. Raw fGSEA p-values for each gene set in each PLS model are depicted inside horizontal bars. Gene sets that were significantly enriched after Bonferroni correction (adjusted p<0.05) are highlighted with boldened horizontal bars.

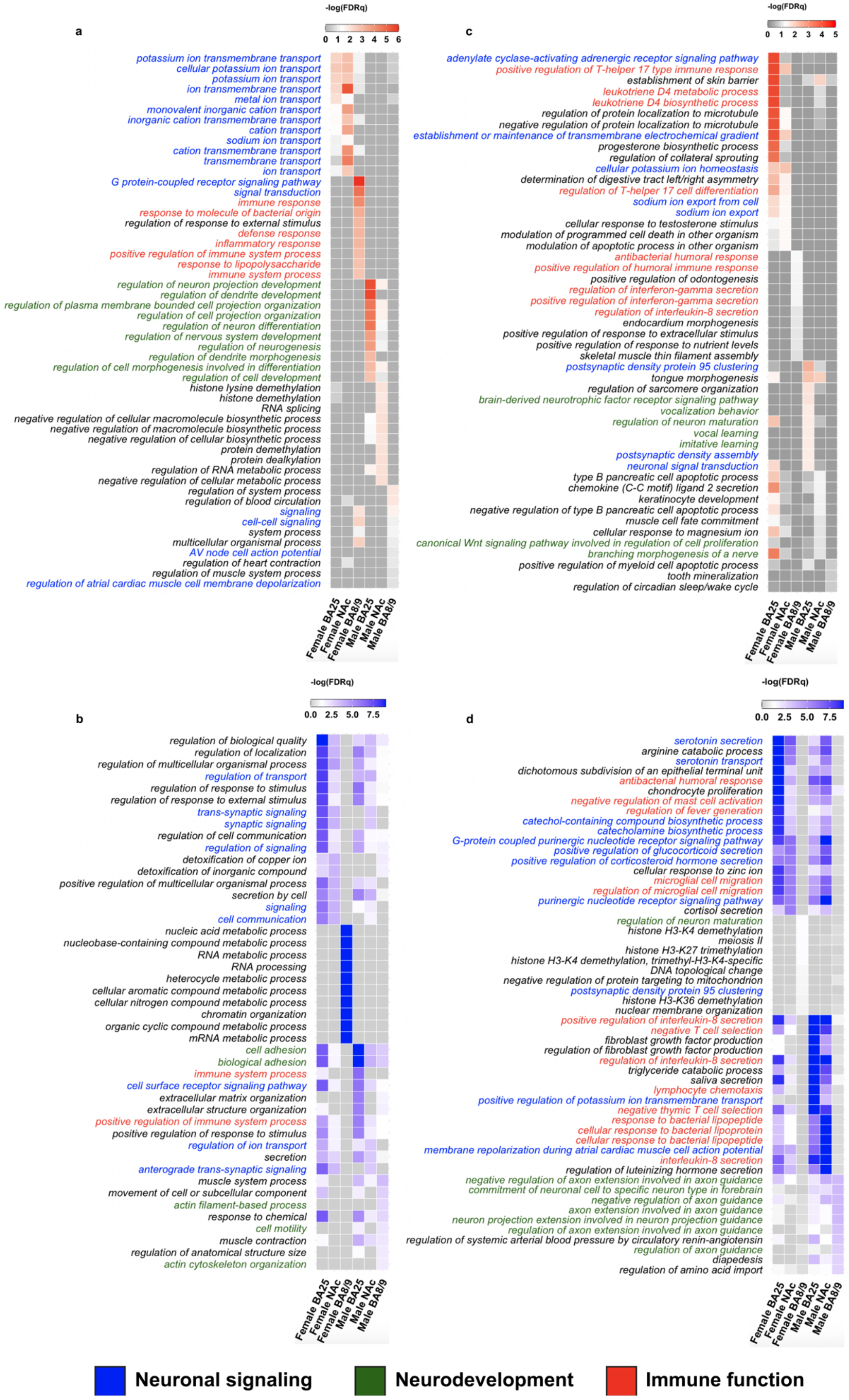

a
-log(FDRq)
0 1 2 3 4 5 6
potassium ion transmembrane transport
cellular potassium ion transport
potassium ion transport
ion transmembrane transport
metal ion transport
monovalent inorganic cation transport
inorganic cation transmembrane transport
cation transport
sodium ion transport
cation transmembrane transport
transmembrane transport
ion transport
G protein-coupled receptor signaling pathway
signal transduction
immune response
response to molecule of bacterial origin
regulation of response to external stimulus
defense response
inflammatory response
positive regulation of immune system process
response to lipopolysaccharide
immune system process
regulation of neuron projection development
regulation of dendrite development
regulation of plasma membrane bounded cell projection organization
regulation of cell projection organization
regulation of neuron differentiation
regulation of nervous system development
regulation of neurogenesis
regulation of dendrite morphogenesis
regulation of cell morphogenesis involved in differentiation
regulation of cell development
histone lysine demethylation
histone demethylation
RNA splicing
negative regulation of cellular macromolecule biosynthetic process
negative regulation of macromolecule biosynthetic process
negative regulation of cellular biosynthetic process
protein demethylation
protein dealkylation
regulation of RNA metabolic process
negative regulation of cellular metabolic process
regulation of system process
regulation of blood circulation
signaling
cell-cell signaling
system process
multicellular organismal process
AV node cell action potential
regulation of heart contraction
regulation of muscle system process
regulation of atrial cardiac muscle cell membrane depolarization
Female BA25
Female NAc
Female BA8/9
Male BA25
Male NAc
Male BA8/9
c
-log(FDRq)
0 1 2 3 4 5
adenylate cyclase-activating adrenergic receptor signaling pathway
positive regulation of T-helper 17 type immune response
establishment of skin barrier
leukotriene D4 metabolic process
leukotriene D4 biosynthetic process
regulation of protein localization to microtubule
negative regulation of protein localization to microtubule
establishment or maintenance of transmembrane electrochemical gradient
progesterone biosynthetic process
regulation of collateral sprouting
cellular potassium ion homeostasis
determination of digestive tract left/right asymmetry
regulation of T-helper 17 cell differentiation
sodium ion export from cell
sodium ion export
cellular response to testosterone stimulus
modulation of programmed cell death in other organism
modulation of apoptotic process in other organism
antibacterial humoral response
positive regulation of humoral immune response
positive regulation of odontogenesis
regulation of interferon-gamma secretion
positive regulation of interferon-gamma secretion
regulation of interleukin-8 secretion
endocardium morphogenesis
positive regulation of response to extracellular stimulus
positive regulation of response to nutrient levels
skeletal muscle thin filament assembly
postsynaptic density protein 95 clustering
tongue morphogenesis
regulation of sarcomere organization
brain-derived neurotrophic factor receptor signaling pathway
vocalization behavior
regulation of neuron maturation
vocal learning
imitative learning
postsynaptic density assembly
neuronal signal transduction
type B pancreatic cell apoptotic process
chemokine (C-C motif) ligand 2 secretion
keratinocyte development
negative regulation of type B pancreatic cell apoptotic process
muscle cell fate commitment
cellular response to magnesium ion
canonical Wnt signaling pathway involved in regulation of cell proliferation
branching morphogenesis of a nerve
positive regulation of myeloid cell apoptotic process
tooth mineralization
regulation of circadian sleep/wake cycle
Female BA25
Female NAc
Female BA8/9
Male BA25
Male NAc
Male BA8/9
b
-log(FDRq)
0.0 2.5 5.0 7.5
regulation of biological quality
regulation of localization
regulation of multicellular organismal process
regulation of transport
regulation of response to stimulus
regulation of response to external stimulus
trans-synaptic signaling
synaptic signaling
regulation of cell communication
regulation of signaling
detoxification of copper ion
detoxification of inorganic compound
positive regulation of multicellular organismal process
secretion by cell
signaling
cell communication
nucleic acid metabolic process
nucleobase-containing compound metabolic process
RNA metabolic process
RNA processing
heterocycle metabolic process
cellular aromatic compound metabolic process
cellular nitrogen compound metabolic process
chromatin organization
organic cyclic compound metabolic process
mRNA metabolic process
cell adhesion
biological adhesion
immune system process
cell surface receptor signaling pathway
extracellular matrix organization
extracellular structure organization
positive regulation of immune system process
positive regulation of response to stimulus
regulation of ion transport
secretion
anterograde trans-synaptic signaling
muscle system process
movement of cell or subcellular component
actin filament-based process
response to chemical
cell motility
muscle contraction
regulation of anatomical structure size
actin cytoskeleton organization
Female BA25
Female NAc
Female BA8/9
Male BA25
Male NAc
Male BA8/9
d
-log(FDRq)
0.0 2.5 5.0 7.5
serotonin secretion
arginine catabolic process
serotonin transport
dichotomous subdivision of an epithelial terminal unit
antibacterial humoral response
chondrocyte proliferation
negative regulation of mast cell activation
regulation of fever generation
catechol-containing compound biosynthetic process
catecholamine biosynthetic process
G-protein coupled purinergic nucleotide receptor signaling pathway
positive regulation of glucocorticoid secretion
positive regulation of corticosteroid hormone secretion
cellular response to zinc ion
microglial cell migration
regulation of microglial cell migration
purinergic nucleotide receptor signaling pathway
cortisol secretion
regulation of neuron maturation
histone H3-K4 demethylation
meiosis II
histone H3-K27 trimethylation
histone H3-K4 demethylation, trimethyl-H3-K4-specific
DNA topological change
negative regulation of protein targeting to mitochondrion
postsynaptic density protein 95 clustering
histone H3-K36 demethylation
nuclear membrane organization
positive regulation of interleukin-8 secretion
negative T cell selection
fibroblast growth factor production
regulation of fibroblast growth factor production
regulation of interleukin-8 secretion
triglyceride catabolic process
saliva secretion
lymphocyte chemotaxis
positive regulation of potassium ion transmembrane transport
negative thymic T cell selection
response to bacterial lipopeptide
cellular response to bacterial lipoprotein
cellular response to bacterial lipopeptide
membrane repolarization during atrial cardiac muscle cell action potential
interleukin-8 secretion
regulation of luteinizing hormone secretion
negative regulation of axon extension involved in axon guidance
commitment of neuronal cell to specific neuron type in forebrain
negative regulation of axon guidance
axon extension involved in axon guidance
neuron projection extension involved in neuron projection guidance
regulation of axon extension involved in axon guidance
regulation of systemic arterial blood pressure by circulatory renin-angiotensin
regulation of axon guidance
diapedesis
regulation of amino acid import
Female BA25
Female NAc
Female BA8/9
Male BA25
Male NAc
Male BA8/9
Neuronal signaling
Neurodevelopment
Immune function

Supplementary Figure 10. Over-representation of Gene Ontologies in Highly Significant PLS-R Models Across Both Sexes. Top 10 most significant Gene Ontology Biological Process terms enriched among the (**a,c**) most positively- and (**b,d**) most negatively-weighted genes from the six PLS models from (**Fig. 4f-g**). FDR q-values representing significance of enrichment of genes from a given GO term in a given loading weight-ranked gene list are plotted in colors, with grey-to-white gradients representing q-values >0.05 and white-to-red or white-to-blue gradients representing significant q-values <0.05. GO enrichment analyses were carried out using two techniques to test for convergent enrichments: GOrilla (http://cbl-gorilla.cs.technion.ac.il/) (**a-b**) and a spatial brain permutation null phenotype[3] (**c-d**). GO terms were manually colored by their involvement in 3 broad categories: neuronal signaling (blue), neurodevelopment (green), and immune function (red).

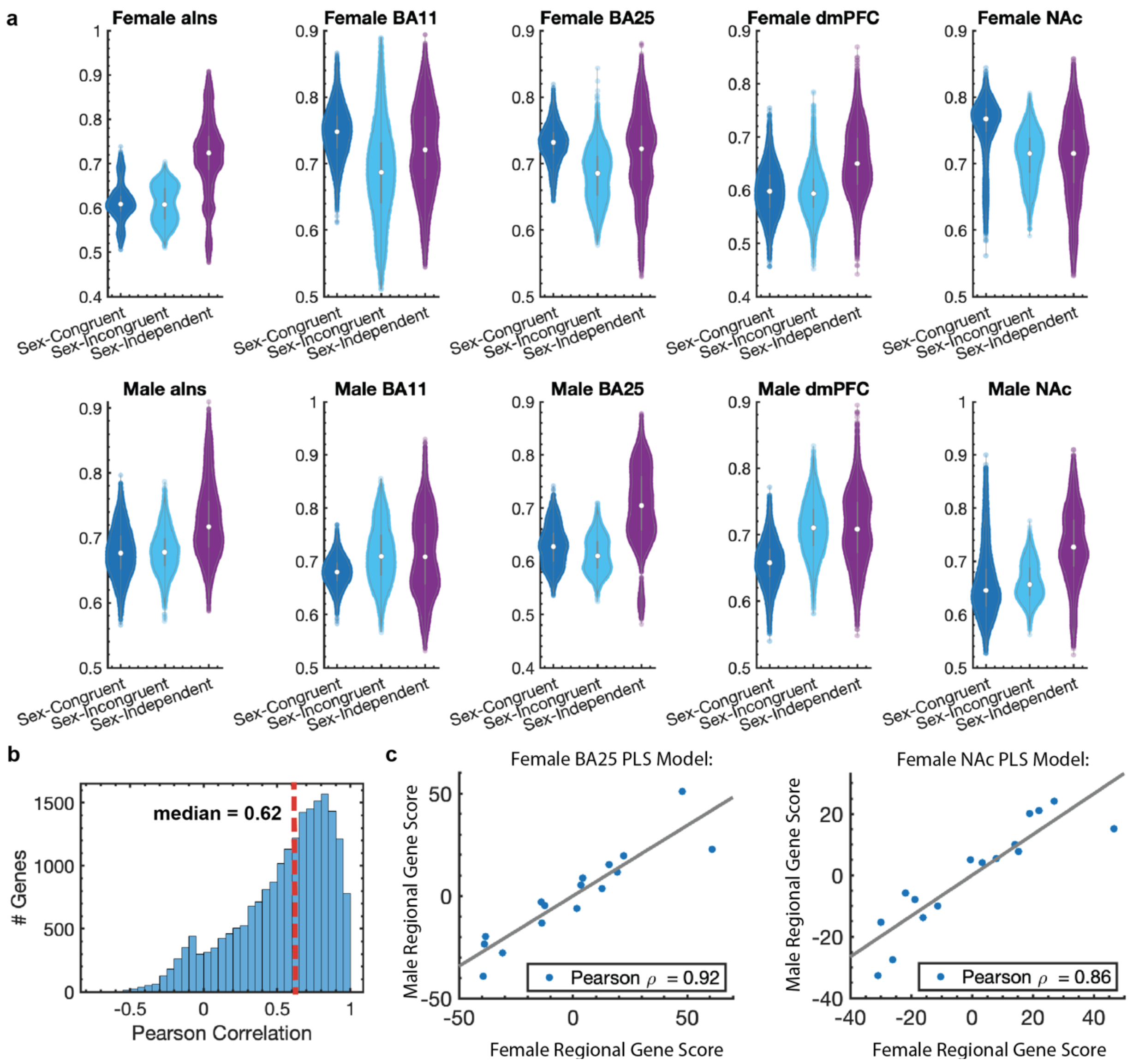

Supplementary Figure 11. Similarities in male and female brain gene expression patterns from Brainspan data. a. Brain gene expression patterns from the same sex do not explain greater variance in depression-related rsFC changes than do brain gene expression patterns from the opposite sex or both sexes. **b.** Correlation of each gene's spatial expression pattern between male and female brain gene expression matrices derived from Brainspan data. 20,287 genes were tested in total. **c.** Gene loading scores based on genes identified by PLS using Allen Human Brain Atlas are similar in male (n=4) and female (n=4) brain gene expression matrices derived from Brainspan data.

	# Subjects	Mean Age ± SD	Mean HAM-D Score ± SD
ThreeD Data Set			
Depressed	371	41.9 ± 11.4	23.6 ± 4.4
Male	148	41.9 ± 11.9	23.2 ± 4.8
Female	223	41.9 ± 11.1	23.8 ± 4.3
Healthy	85	35.1 ± 13.0	N/A
Male	33	33.3 ± 12.3	N/A
Female	52	36.2 ± 13.4	N/A
fc1000 Data Set			
Healthy	97	43.6 ± 16.2	N/A
Male	46	44.0 ± 14.0	N/A
Female	51	43.2 ± 18.1	N/A

Supplementary Table 1: Subject Profile. Subjects came from four scanner sites; “ThreeD” (Toronto) and three sites from the “fc1000” dataset (“ICBM”, “NewYork_a”, and “Cleveland CCF”). Number of subjects included in rsfMRI analysis, mean age, and mean Hamilton Depression Rating Score with standard deviations are shown for subjects grouped by sex and diagnosis from each dataset.

Supplementary References

1 Kang, H. J. *et al.* Spatio-temporal transcriptome of the human brain. *Nature* **478**, 483-489, doi:10.1038/nature10523 (2011).

2 Gershoni, M. & Pietrokovski, S. The landscape of sex-differential transcriptome and its consequent selection in human adults. *BMC Biol* **15**, 7, doi:10.1186/s12915-017-0352-z (2017).

3 Fulcher, B. D., Arnatkeviciute, A. & Fornito, A. Overcoming false-positive gene-category enrichment in the analysis of spatially resolved transcriptomic brain atlas data. *Nat Commun* **12**, 2669, doi:10.1038/s41467-021-22862-1 (2021).
