# Supplementary material for: Regional gene expression signatures are associated with sex-specific functional connectivity changes in depression
Source: Nat Commun. 2022 Sep 28;13:5692. doi: 10.1038/s41467-022-32617-1 (PMC9519925; doi:10.1038/s41467-022-32617-1)
Supplement: Supplementary file 2 — Reporting Summary [file 41467_2022_32617_MOESM2_ESM.pdf]

nature research

Corresponding author(s): Conor Liston
Last updated by author(s): Jul 29, 2022

Reporting Summary

Nature Research wishes to improve the reproducibility of the work that we publish. This form provides structure for consistency and transparency in reporting. For further information on Nature Research policies, see our Editorial Policies and the Editorial Policy Checklist.

Statistics

For all statistical analyses, confirm that the following items are present in the figure legend, table legend, main text, or Methods section.

n/a	Confirmed	
☐	☒	The exact sample size (n) for each experimental group/condition, given as a discrete number and unit of measurement
☐	☒	A statement on whether measurements were taken from distinct samples or whether the same sample was measured repeatedly
☐	☒	The statistical test(s) used AND whether they are one- or two-sided<br>*Only common tests should be described solely by name; describe more complex techniques in the Methods section.*
☐	☒	A description of all covariates tested
☐	☒	A description of any assumptions or corrections, such as tests of normality and adjustment for multiple comparisons
☐	☒	A full description of the statistical parameters including central tendency (e.g. means) or other basic estimates (e.g. regression coefficient) AND variation (e.g. standard deviation) or associated estimates of uncertainty (e.g. confidence intervals)
☐	☒	For null hypothesis testing, the test statistic (e.g. F, t, r) with confidence intervals, effect sizes, degrees of freedom and P value noted<br>*Give P values as exact values whenever suitable.*
☒	☐	For Bayesian analysis, information on the choice of priors and Markov chain Monte Carlo settings
☒	☐	For hierarchical and complex designs, identification of the appropriate level for tests and full reporting of outcomes
☐	☒	Estimates of effect sizes (e.g. Cohen's d, Pearson's r), indicating how they were calculated

Our web collection on statistics for biologists contains articles on many of the points above.

Software and code

Policy information about availability of computer code

Data collection	No software was used for data collection
Data analysis	Matlab 2019a<br>R 3.6.2<br>org.Hs.eg.db version 3.11.4; freely available for download in R through Bioconductor.<br>CONN toolbox: available for download at <https://web.conn-toolbox.org/><br>Midnight Scan Club Codebase: available for download at <https://github.com/MidnightScanClub/MSCcodebase><br>'spls' package by Joao M. Monteiro; available for download at <https://github.com/jmmonteiro/spls><br>'rotate-parcellation' package by Frantisek Vasa; available for download at <https://github.com/frantisekvasa/rotate_parcellation><br>'fGSEA' package by Alexey Sergushichev; available for download in R through Bioconductor.<br>'qctool version 2'; available for download at <https://www.well.ox.ac.uk/~gav/qctool_v2/>

For manuscripts utilizing custom algorithms or software that are central to the research but not yet described in published literature, software must be made available to editors and reviewers. We strongly encourage code deposition in a community repository (e.g. GitHub). See the Nature Research guidelines for submitting code & software for further information.

Data

Policy information about availability of data

All manuscripts must include a data availability statement. This statement should provide the following information, where applicable:

- Accession codes, unique identifiers, or web links for publicly available datasets
- A list of figures that have associated raw data
- A description of any restrictions on data availability

Data Availability Statement

There were no new raw data generated in this study. All preprocessing and analysis were performed on pre-existing data sets as described in the Methods section. The Three-D study fMRI and UK Biobank data are available under restricted access to protect patient privacy. Access can be obtained upon reasonable request by contacting the authors of this paper who manage each dataset: Dr. Jonathan Downar for the Three-D data set, and Dr. Andrew Macintosh for the UK Biobank dataset. UK Biobank access can also be obtained via a centralized application process at <www.ukbiobank.ac.uk/enable-your-research/apply-for-access>. The Allen Human Brain Atlas and Brainspan datasets are freely available and may be downloaded at <http://human.brain-map.org/static/download> and <https://www.brainspan.org/static/download.html>, respectively. Genome Tissue Expression Database version 8 significant cis-QTL variant-gene associations; freely available for download at <https://www.gtexportal.org/home/datasets>. The gene ranking results of partial least squares regression analyses applied to AHBA and Brainspan data are made available in Supplementary Table 2, and Gene Ontology terms derived from those ranked gene lists are made available in Supplementary Table 3.

Code Availability Statement

The code used for machine learning analyses described in this manuscript is made available along with example data in the following Github repository: https://github.com/AlexTalishinsky/Sex_Dimorphic_Depression_rsFC_Gene_Correlates
The original version used for the manuscript is citable via the Zenodo repository at the following DOI: <10.5281/zenodo.6825246>. No novel algorithms were used in the analyses therein.

Field-specific reporting

Please select the one below that is the best fit for your research. If you are not sure, read the appropriate sections before making your selection.

[x] Life sciences [ ] Behavioural & social sciences [ ] Ecological, evolutionary & environmental sciences

For a reference copy of the document with all sections, see nature.com/documents/nr-reporting-summary-flat.pdf

Life sciences study design

All studies must disclose on these points even when the disclosure is negative.

Sample size	This was a retrospective analysis of two datasets. Most analyses were conducted in fMRI data acquired through the THREE-D study, in which the sample size was powered to evaluate the non-inferiority of a 3-minute transcranial magnetic stimulation protocol (intermittent theta burst) compared to a commonly used 37-minute (10-Hz) protocol. Key results and predictions were replicated and tested in fMRI data from the UK Biobank. In both cases, we used all data available to us in these two datasets. Because the sample sizes were pre-determined in this retrospective analysis, we did not perform power analyses to determine sample sizes prior to the study.
Data exclusions	Subjects were excluded if they had <5 minutes of resting-state fMRI data after scrubbing motion-contaminated frames. Subjects were also excluded based on age (eg age<18; see Methods for details) so that case and control groups did not show significant differences in age.
Replication	Findings of sex-specific default mode rsFC changes associated with depression (Fig. 1) were replicated in a separate subject cohort scanned at a different site under different parameters (Fig. 2).<br>Findings based on multivariate models trained using transcriptomic data from the Allen Human Brain Atlas (Fig. 4) were partially replicated using transcriptomic data from the Brainspan database (Supplementary Figs. 7,8,10).
Randomization	This was a retrospective analysis of the effect of depression and sex on functional connectivity. There was no experimental intervention, so randomization to an intervention was not applicable in this study.
Blinding	This was a retrospective analysis of the effect of depression and sex on functional connectivity. There was no experimental intervention, so blinding of the experimenter or participant to an assigned intervention was not applicable in this study.

Reporting for specific materials, systems and methods

We require information from authors about some types of materials, experimental systems and methods used in many studies. Here, indicate whether each material, system or method listed is relevant to your study. If you are not sure if a list item applies to your research, read the appropriate section before selecting a response.

Materials & experimental systems

n/a	Involved in the study	
x		Antibodies
x		Eukaryotic cell lines
x		Palaeontology and archaeology
x		Animals and other organisms
	x	Human research participants
x		Clinical data
x		Dual use research of concern

Methods

n/a	Involved in the study	
x		ChIP-seq
x		Flow cytometry
	x	MRI-based neuroimaging

Human research participants

Policy information about studies involving human research participants

Population characteristics

553 subjects were used for our primary rsfMRI analyses. 371 subjects (223 female) had severe treatment-resistant unipolar depression, while the other 182 (103 female) were healthy controls with no known history of psychiatric disease. Mean age of all subjects was 41.1 (standard deviation = 12.9), and mean age did not differ significantly between depressed and healthy subjects. Genotypic information was not available in our subject cohort.

rsfMRI and survey-response data from 5,138 UK Biobank subjects was also used to reproduce results from our initial cohort of 553 subjects. Full description of subject parameters is available in the Methods section, Supplementary Table 1, and at <https://www.ukbiobank.ac.uk/>.

Recruitment

This study was performed on existing data sets and did not include any further recruitment of subjects.

Ethics oversight

All participants provided informed consent, and research protocols were approved by Institutional Review Boards at the Center for Addiction and Mental Health and the University Health Network, Toronto, and the UK Biobank Research Ethics Committee (reference: 11/NW/0382).

Note that full information on the approval of the study protocol must also be provided in the manuscript.

Magnetic resonance imaging

Experimental design

Design type

Resting-state.

Design specifications

Subjects were scanned at rest and instructed to close their eyes.

Behavioral performance measures

None.

Acquisition

Imaging type(s)

Functional

Field strength

3T for all subjects

Sequence & imaging parameters

Toronto subjects: For each participant, two datasets were acquired: a high resolution, T1-weighted fast spoiled gradient-echo structural dataset (TE = 12 ms, flip angle = 20°, 116 sagittal slices, thickness = 1.5 mm, no gap, 256 x 256 matrix, FOV = 240 mm), and a 10-minute resting-state functional MRI dataset in the eyes closed condition (T2*-weighted EPI, TE = 30 ms, TR = 2000 ms, flip angle = 85°, 32 axial slices, thickness = 5 mm, no gap, 64x64 matrix, FOV = 220mm).

fc1000 subjects: full parameters can be found online at <https://www.nitrc.org/frs/?group_id=296>. Subjects used in our study were from the "NewYork_a", "Cleveland CCF", and "ICBM" sites. Briefly, scanning parameters for these sites were: TR = 2000, 2000, and 2800 ms respectively; 23, 39, and 31 slices, respectively; 128, 192, and 127 volumes, respectively; total scan durations were 6.4 minutes, 5.93 minutes, and 12.81 minutes , respectively.

UK Biobank subjects: full parameters can be found online at <https://www.fmrib.ox.ac.uk/ukbiobank/>. Briefly, TR = 735 ms, TE = 39 ms, 64 slices, slice thickness = 2.40 mm, total scan duration = 6 minutes.

Area of acquisition

Whole-brain scan was used for all subjects

Diffusion MRI ☐ Used ☒ Not used

Preprocessing

Preprocessing software	FSL version 6.0. Specific functions and parameters used are defined below.
Normalization	T1 anatomical volumes in the Toronto and fc1000 samples were cropped to a smaller field of view (150mm in z plane) using FSL's automated robustfov tool and aligned to the 2mm MNI atlas template using a rigid, 6-degrees-of-freedom (DOF) FLIRT transformation. A non-linear transformation between the ACPC aligned T1-weighted anatomical image and MNI atlas (2mm) was estimated using FNIRT. A brain extraction was then performed using a binarized MNI brain mask, transformed from atlas space into native image space using an inverse transformation calculated using FSL's invwarp tool. For each subject, functional data were co-registered to the ACPC-aligned T1-weighted anatomical image using FSL's epi_reg program and transformed into atlas space using the non-linear transformation defined above in the T1 anatomical data.
Normalization template	2mm MNI atlas template (group standardized space).
Noise and artifact removal	Functional data was denoised using the aCompCor strategy110 implemented in the CONN toolbox (version 17.0; https://web.conn-toolbox.org). Denoising steps included linear de-trending and nuisance regression (5 principle components from eroded white matter and cerebrospinal fluid masks from the aforementioned tissue segmentation; 6 motion parameters and first-order temporal derivatives; and point-regressors to censor time points with mean frame-wise displacement > 0.2mm). Residual time-series were band-pass filtered (0.01 Hz < f < 0.1 Hz) after regression to avoid reintroduction of nuisance-related variation in the time-series.
Volume censoring	Temporal masks were created to flag motion-contaminated frames for scrubbing. High-motion volumes were identified by framewise displacement (FD) calculated as the sum of absolute values of the differentials of the three translational motion parameters and three rotational motion parameters. 11 TRD subjects and 2 HC subjects with <5 minutes of uncontaminated resting state data were excluded from further analysis.

Statistical modeling & inference

Model type and settings	N/A: general linear modeling was not used to make voxel- or cluster- based inferences. Resting state data was analyzed via group comparisons and via elastic-net general linear modeling, described briefly below in "Multivariate modeling and predictive analysis" and described fully in the Methods section.
Effect(s) tested	Effect of depression on resting state functional connectivity was tested using two-way factorial ANOVA and two-tailed post-hoc t-tests, as described fully in the Methods section. For all analyses, the figures and figure legends report the test statistic and P value, as well as the sample size, from which the degrees of freedom can be readily derived.

Specify type of analysis: ☐ Whole brain ☐ ROI-based ☒ Both

Anatomical location(s)	Voxels were parcellated into brain ROIs using the 180-region Glasser parcellation. Parcels defined to be within transcriptional regions of interest based on a previously published report were grouped into five seed ROIs (Fig. 3).
Statistic type for inference (See Eklund et al. 2016)	N/A: resting-state data was used and voxels were grouped into pre-defined ROIs according to the previously published and validated Glasser parcellation. Mindful of concerns about false positives due to cluster-based corrections for multiple comparisons, we used established permutation testing frameworks to test for statistical significance (Figs. 3-5) and for mass univariate tests (Figs. 1-2, 6), we used a more stringent brain-wide FDR correction without any cluster-based inferences.
Correction	FDR correction was performed for all p-values resulting from ANOVA or t-test analyses, using the Matlab function 'mafdr'.

Models & analysis

n/a	Involved in the study
☐	☒ Functional and/or effective connectivity
☒	☐ Graph analysis
☐	☒ Multivariate modeling or predictive analysis

Functional and/or effective connectivity	Fisher-Z-transformed Pearson correlations between regional BOLD time series were used to represent functional connectivity.
Multivariate modeling and predictive analysis	We used two multivariate models in this study.<br>1. Elastic-net regularized general linear model: Full description in Methods. Briefly, we modeled depression diagnostic status in 217-subject subsamples (300 iterations) using resting state connectivity features as independent predictor variables. Modeling was performed by inputting predictor and response variables into the Matlab function, 'lassoglm'. Alpha value was set to 0.5, and models were generated at over 75 different lambda values per the default 'lassoglm' functionality. Feature extraction and dimension reduction were not performed. Model performance was evaluated by measuring area under the ROC curve using the Matlab function, 'perfcurve'. Data in the test set in each iteration were strictly independent from all aspects of model training.<br>2. Partial least squares regression model: Full description in Methods. Briefly, we modeled seed-based rsFC changes associated with depression using brain regional gene expression of 21,120 genes as predictor variables in our main analysis. PLS-R modeling was performed using the 'spls' package by J.M. Monteiro, available for download at <https://github.com/jmmonteiro/spls>. Feature extraction and dimension

reduction was not performed. Model performance was evaluated using two validated null permutation approaches for significance testing of PLS-R components, described fully in the Methods. Furthermore, relevant genes highlighted by our models were validated by testing for linear relationships between inferred gene expression levels in nervous tissue and rsFC in depressed male and female UK Biobank subjects (see Methods for additional details).
